# Congenital Anomalies of the Kidney and Urinary Tract in Down Syndrome: Prevalence, Phenotypes, Genetics and Clinical Management

**DOI:** 10.3390/genes16030245

**Published:** 2025-02-20

**Authors:** Mirela Leskur, Dario Leskur, Sandra Marijan, Luka Minarik, Bernarda Lozić

**Affiliations:** 1 Department of Biochemistry and Medical Chemistry, University of Split School of Medicine, 21000 Split, Croatia; sandra.marijan@mefst.hr; 2 Department of Pharmacy, University of Split School of Medicine, 21000 Split, Croatia; 3 Institute of Emergency Medicine, 10000 Zagreb, Croatia; 4 Department of Paediatrics, University of Split School of Medicine, 21000 Split, Croatia; blozic@kbsplit.hr; 5 Department of Pediatric Disease, Division of Haematology, Oncology, Clinical Immunology and Genetics, University Hospital of Split, 21000 Split, Croatia

**Keywords:** down syndrome, CAKUT, Trisomy 21, kidney, urinary tract, congenital anomalies, glomerulopathies, genetics

## Abstract

Down syndrome (DS), the most common survivable autosomal aneuploidy, is associated with a high prevalence of congenital anomalies of the kidney and urinary tract (CAKUT), significantly increasing the risk of chronic kidney disease (CKD). This review examines the diversity of CAKUT phenotypes reported in individuals with DS, focusing on anomalies affecting the kidney, ureter, bladder, and urethra. According to available literature, hydronephrosis is the most common renal anomaly, often secondary to other CAKUT phenotypes, followed by renal hypoplasia and glomerulocystic disease. Furthermore, obstructive uropathies are also frequent but usually lack detailed characterization in the literature. Key features of CAKUT in DS, including reduced kidney size, renal cystic diseases, acquired glomerulopathies, reduced nephron number, and immature glomeruli heighten the risk of CKD. Also, early detection of lower urinary tract dysfunction (LUTD) is critical to prevent progressive upper urinary tract damage and CKD. Despite the prevalence of CAKUT in DS, reported between 0.22% and 21.16%, there is a lack of standardized diagnostic criteria, consistent terminology, and extended follow-up studies. Systematic screening from infancy, including regular renal monitoring via urinalysis and ultrasound, plays a critical role in the timely diagnosis and intervention of CAKUT. To further enhance diagnostic accuracy and develop effective therapeutic strategies, increased awareness and focused research into the genetic factors underlying these anomalies are essential. Moreover, a multidisciplinary approach is indispensable for managing CAKUT and its associated complications, ultimately ensuring better long-term outcomes and an improved quality of life for individuals with DS.

## 1. Introduction

Down syndrome (DS), also known as Trisomy 21, is the most common survivable autosomal aneuploidy, with a prevalence of 10.1 per 10,000 live births in Europe, which would be around 21.7 per 10,000 live births without elective terminations [1]. It is predominantly caused by an error in meiotic divisions during oocyte formation, leading to a supernumerary chromosome 21 [2]. This genetic anomaly results in a collection of clinical features known as DS, which is among the most genetically complex conditions compatible with human survival post-term. DS is characterized by intellectual disability and various congenital malformations that can affect multiple organ systems, including the urinary tract and kidneys [3].

Advances in treatment have significantly increased the life expectancy of individuals with DS to over 50 years. As a result, 4.5% of DS patients are now affected by severe chronic kidney disease (CKD), typically occurring during puberty with an unclear etiology. Consequently, the impacts of associated anomalies on the long-term care of these patients are becoming increasingly important. However, congenital anomalies of the kidney and urinary tract (CAKUT) have been relatively underexplored compared to other congenital anomalies present in DS [4].

CAKUT encompasses all malformations resulting in abnormal development of the kidneys and urinary system. These anomalies can vary widely regarding location, severity, and the specific structures they affect. Major defects, such as kidney agenesis and malformed kidneys, occur early in development, while lesser defects, like obstruction, vesicoureteral reflux (VUR), or posterior urethral valves (PUVs), arise later. Despite contributing to over 50% of pediatric CKD cases, the prevalence of CAKUT is very low in the general population (<1% of live births) [5]. Current reviews and guidelines on DS only marginally address renal and urinary tract anomalies, highlighting the need for further study in this area.

In this article, we offer a comprehensive review of published literature on CAKUT documented in individuals with DS. Additionally, we aim to shed light on the prevalence, genetic factors, diagnostic methods, and medical and surgical management of CAKUT along with lower urinary tract dysfunction (LUTD) and kidney function in individuals with DS, emphasizing the need for increased awareness and research in this area.

## 2. Autopsy and Small Studies

The distinctive features of DS were initially documented by John Laughton Down in 1866. It was not until almost a century later, in 1960, that Berg et al. reported the first instances of DS accompanied by renal and urological malformations [6,7]. The first studies to report CAKUT in DS patients were based on autopsy examinations, which provided post-mortem analyses of renal and urinary tract abnormalities. Other than autopsy studies, this section includes small studies such as retrospective chart reviews, case studies, cross-sectional studies, and retrospective cohort studies (Table 1). In the following paragraphs, we will review selected studies chronologically.

Berg et al. (1960) were the first to report renal and urological malformations in DS, examining autopsy records of 141 children and identifying severe CAKUT phenotypes in 3.5% of cases, including renal agenesis, renal hypoplasia, and horseshoe kidney [7].

Egli and Stalder (1973) conducted a specific examination of data and/or autopsies of 103 DS cases and other trisomies, revealing a 6.7% frequency of CAKUT. Their study, although lacking systematic evaluation and excluding renal status assessments, identified hydronephrosis, hydroureter, large bladder, and ureteral stenosis, along with the first report of renal cysts. They also concluded that no specific CAKUT phenotype correlated with any particular chromosome [9].

In 1991, Ariel et al. specifically addressed the lack of attention to CAKUT in DS through a thorough review of 124 autopsy reports. They documented various CAKUT phenotypes, finding obstructive uropathy in 6.5% of cases. The study noted degrees of hydronephrosis, hydroureters, cystic dysplasia, and trabeculated bladders, all associated with obstructive defects. However, the absence of control subjects limited the contextualization of their findings. The exact number of DS individuals with CAKUT phenotypes remains unknown, making it difficult to determine prevalence. Nevertheless, this study was significant as it was the first to specifically investigate CAKUT in DS [10].

A small retrospective study by Subrahmanyam and Mehta (1995) concluded that the incidence of renal anomalies was not increased in DS, based on a clinical evaluation of 54 DS subjects aged 2 months to 24 years, excluding fetuses, stillbirths, and infants. Only one child (1.9%) had a CAKUT defect, which was urethral stenosis [11].

In 2004, Malaga et al. conducted a cross-sectional study on 69 DS patients, the largest at the time, to specifically investigate young DS patients for renal disease. They found minor defects such as bladder hypertrophy, ectopic kidney, pyelectasis, and kidney hypoplasia in 7.3% of the subjects, suggesting a similar incidence of renal anomalies to the general population [13].

A brief report by Jain et al. (2014) suggested a high prevalence of CAKUT in 40 DS children. However, the study did not provide detailed data, only noting that ultrasonography detected hydronephrosis in 20% of cases, with additional mentions of hydroureter, renal hypoplasia, VUR, and ureteropelvic junction obstruction (UPJO) without specifying their frequencies [17]. 

Postolache et al. (2022) identified CAKUT phenotypes in 7 out of 49 DS children (14.3%). They found hydronephrosis with pyelocaliceal dilatation in two children, pyelectasis in one child, and reduced cortico-medullary differentiation in two children. No kidney or urinary tract anomalies were detected in the control group. Among the 27 DS participants with a history of congenital heart defects (CHD), no association was found between CHD and kidney function markers (eGFR, albuminuria, or kidney length) [4].

**Section summary:** Small autopsy-based investigations and targeted clinical studies offer valuable, detailed insights into specific renal and urological anomalies in this population. Early autopsy studies identified severe CAKUT phenotypes, while subsequent clinical studies report varying frequencies of milder anomalies, with indications of a potentially higher prevalence of obstructive uropathies. However, these findings are complicated by methodological variations across studies, including differences in sample selection and diagnostic approaches. Additionally, many studies lack detailed descriptions and precise numerical data on CAKUT prevalence in DS patients, further obscuring the true frequency of these anomalies. To advance understanding in this area, particularly concerning the nature and incidence of CAKUT, further research with standardized diagnostic criteria and consistent study designs is warranted.

## 3. Prevalence of CAKUT in DS and Population-Based Studies

Understanding the prevalence of CAKUT in DS is crucial for enhancing early detection, improving patient care, guiding resource allocation, informing research, and ultimately improving the health and well-being of individuals with DS [19]. Major CAKUT anomalies significantly impact morbidity, mortality, and the provision of healthcare services [5,19]. Therefore, it is essential to determine the prevalence of these congenital anomalies in babies with DS. Estimates from population-based studies place the prevalence of CAKUT between 0.04% and 1% of the general population, with genetic disorders contributing to 10–20% of these cases [20,21,22].

To calculate prevalence accurately, population-based studies are generally considered the best method. Several such studies have examined the occurrence of birth defects in infants with DS and compared them with controls [22]. However, these studies often face several limitations.

A key issue with older studies is the reliance on phenotypic descriptions to classify a child as having DS due to the variable availability of karyotyping. Karyotyping for DS diagnosis, introduced shortly after Dr. Jérôme Lejeune’s discovery in 1959, revolutionized chromosomal analysis. However, its accessibility varied across regions and healthcare facilities, influenced by technology adoption and resource availability [3,23].

Furthermore, the diagnostic capabilities of older ultrasound technology were limited, complicating comparisons with newer studies. Additionally, some studies aggregate data from multiple registries with different methods of ascertainment, leading to heterogeneity [24]. Restrictions on the number of anomalies reported per case can result in significant underreporting of CAKUT.

As previously mentioned, CAKUT encompasses a diverse group of structural abnormalities of the kidneys and urinary system, which vary widely in severity depending on their location and the specific structures affected [5]. Most population-based studies tend to concentrate on major anomalies such as renal agenesis, often overlooking other conditions and not specifying the detailed nature of less prominent anomalies. 

A major limitation in most population-based studies lies in the lack of data on stillbirths, miscarriages, and Terminations of Pregnancy for Fetal Anomaly (TOPFAs), focusing primarily on conceptuses that survived to term. However, the risk of spontaneous miscarriage is higher in pregnancies affected by DS compared to those that are unaffected. Reports from England and Wales estimate the average miscarriage rate between chorionic villus sampling (at 10 weeks gestation) and term is 32% (95% CI: 26–38), and the average miscarriage rate between amniocentesis and term is 25% (95% CI: 21–31) [25]. Additionally, the risk of stillbirth in DS pregnancies without other major congenital anomalies was assessed to be 58 times greater compared to the general population risk [26]. Miscarriages and stillbirths in DS are often linked to major anomalies incompatible with life, including several CAKUT phenotypes, which in turn leads to the underreporting of CAKUT if not taken into consideration. 

De Graf et al. estimated that the rate of TOPFAs for DS in Europe between 2011 and 2015 averaged 54%, ranging from 0% in Malta to 83% in Spain [1]. The proportion of TOPFAs has increased continually since 2000, which authors linked to an increase in the use of first-trimester screening tests in Europe. Before women undergo a TOPFA, only the more severe congenital anomalies are searched for and reported following a fetal ultrasound. In contrast, less severe congenital anomalies are not as easily detected by ultrasound and are only reported after birth, which also leads to a seemingly lower prevalence of CAKUT if a study has data on TOPFAs [27]. 

Moreover, these studies typically have a short reporting period (mostly up to the first year of life), while less severe CAKUT phenotypes may manifest later in life [28]. In the following paragraphs, we will review all available population-based studies chronologically (Table 2).

The first population-based study to include CAKUT phenotypes was a multinational cohort study conducted in 1996. Kallen et al. gathered data on 5581 infants with DS from three registries (Central-East France Registry, Italy: IPIMC Registry and The Swedish Registry) focusing on the occurrence of 23 major malformations (International Clearinghouse for Birth Defects Monitoring Systems (ICBDMS) diagnostic coding). These malformations included only kidney agenesis/dysgenesis and hypospadias from the CAKUT range. Notably, the karyotype was unknown in approximately 30% of infants, potentially affecting the accuracy of findings due to possible misdiagnoses. Furthermore, the study did not include TOPFAs, which could have provided valuable insights into CAKUT across different severities. The authors acknowledged that their reported rates of malformations might be conservative, as there could have been underreporting by cytogenetic laboratories. Regarding CAKUT, their findings indicated a prevalence of 0.2%, with the occurrence of hypospadias found to be compatible with a normal rate and a twofold increased risk of kidney agenesis/dysgenesis that was not statistically significant. Defect rates of the control population were calculated from the ICBDMS report [30,36].

Torfs and Christianson (1998) conducted a comprehensive study using 10 years of data from the California Birth Defects Monitoring Program (CBDMP), focusing on structural defects (diagnostic coding: BPA6) in 2894 confirmed cases of DS and a control population. The CBDMP gathers data from various sources, including obstetric, nursery, pediatric, pathology, and cytogenetic laboratory records, covering over half of all births in California over a decade. Notably, stillbirths and TOPFAs were excluded, and infants were followed up until they reached one year of age. The authors highlighted challenges in classifying CAKUT, noting frequent misclassification issues. Therefore, their analysis focused on anomalies less prone to misclassification, such as renal agenesis, horseshoe kidney, and general obstructive defects (including hydronephrosis). Their findings revealed a prevalence rate of 3.2% for CAKUT. Horseshoe kidneys showed a significant association with DS (risk ratio (RR) 11.7, 0.001 < *p* < 0.05) along with obstructive defects, which also demonstrated a high RR of 14.2 (*p* < 10^−6^), whereas renal agenesis did not show a statistically significant association. Hypospadias and epispadias also occurred more frequently in DS patients (RR 5.3, *p* < 10^−6^). The study did not find an increased risk of renal agenesis or bladder exstrophy in DS cases. The authors speculated that the lack of association might be due to severe defects leading to miscarriages or abortions, as the study did not include stillbirths or TOPFAs, potentially resulting in an underestimation of CAKUT prevalence. The authors emphasized the study’s strengths, including uniform data collection methods and cytogenetic testing, which contributed to its robustness compared to previous research efforts [31].

In 2007, Cleves et al. conducted a study using national hospital discharge datasets, namely the Nationwide Inpatient Sample (NIS) and Kids Inpatient Database (KID), to examine the prevalence of 45 structural birth defects (diagnostic coding: ICD-9-CM) among newborns with DS and a control population over a 10-year period. This study marked the first utilization of large, nationally representative datasets covering approximately 8 million births. Despite its strengths, such as broad population coverage, the study faced limitations, including incomplete hospital records, variability in diagnostic and coding practices, and the absence of follow-up validation procedures. The study revealed that 2.2% of discharged newborns with DS had CAKUT malformations, with 3.6 (95% CI:3.1–4.2) times higher odds of developing CAKUT compared to infants without DS. Among these malformations, obstructive defects were the most common, with an odds ratio (OR) of 5.8 (95% CI:4.9–6.9). Hypospadias was also associated with DS with an OR of 2.1 (95% CI:1.7–2.6). However, there was no statistically significant difference in rates of renal agenesis and hypoplasia between the two populations. The authors acknowledged several shortcomings, such as the lack of information on whether karyotyping was performed to confirm DS and the exclusion of data on stillbirths or TOPFAs. This exclusion might have led to an underestimation of the number and types of CAKUT associated with DS. Furthermore, the study’s inclusion criteria focused on easily recognizable conditions, omitting conditions requiring extensive imaging for detection. The authors also noted that discharge data typically underestimate rates reported by state surveillance systems that include TOPFAs, potentially by up to 40%. Despite these limitations, the Cleves et al. study provided valuable insights into the prevalence and types of structural birth defects among infants with DS using extensive national datasets [32].

A research team based in Strasbourg has conducted multiple studies focusing on the epidemiology of DS and its associated malformations. Their investigations included all registered newborns, as well as stillbirths and TOPFAs, with surveillance extending until the age of two. Each DS case underwent karyotyping, supplemented by ultrasonographic examination in 97.7% of cases. By 1987, their registry had documented 139 DS cases, revealing that 2.8% exhibited some form of CAKUT [29]. By 2008, the registry expanded to include 728 DS cases, with CAKUT emerging as the fourth most common anomaly with a prevalence of 3.94% and obstructive defects constituting 50% of these cases [34]. The researchers acknowledged limitations in their study: major anomalies within the same organ system were counted as a single defect, and there were no control subjects to compare with. Despite this, their observations indicated an increased frequency of CAKUT in DS cases compared to previous studies [29,34].

In 2009, Kupferman et al. published the first retrospective cohort study specifically examining the occurrence of CAKUT in a population with DS and a control population. They utilized 12 years of data from the New York State Congenital Malformation Registry (NYS-CMR), which mandates reporting of any birth defects (diagnostic coding: ICD-9-CM) up to the age of 2 years. The study revealed that the prevalence of CAKUT in DS was 3.2%, with an OR of 4.5 (95% CI:3.8–5.4). The authors also identified a significantly increased risk of specific CAKUT phenotypes, including anterior urethral obstruction, cystic dysplastic kidney, hydronephrosis, hydroureter, PUVs, and renal agenesis. Additionally, prune belly syndrome, characterized by an enlarged bladder, among other symptoms, was found to be approximately 12 times more common in the DS population compared to the general population (OR 11.9, 95% CI 1.6–85.4). Despite these findings, the study had limitations that likely led to an underestimation of CAKUT prevalence. Notably, renal hypoplasia, which has been reported in DS, was not included in their analysis. The study did not include stillbirths or TOPFAs. Furthermore, malformations diagnosed on an outpatient basis were not well-reported, contributing to potential underreporting [19]. 

A population-based study by Rankin et al. (2012) examined data on DS subjects obtained from the UK Northern Congenital Abnormality Survey (NorCAS; diagnostic coding: ICD-10) along with hospital and national mortality records and found that CAKUT anomalies were the third most occurring abnormalities with a prevalence of 1.1%. Unfortunately, hydronephrosis was the only phenotype specified with a prevalence of 0.6%. This study included miscarriages, TOPFAs, stillbirths, and live births. What was interesting was that only 37% of cases with CAKUT resulted in live births. This study did not compare the prevalence of CAKUT between DS and non-DS populations, nor could it calculate rates and risks between those populations, as it did not include any controls. Instead, this study only focused on predictors of survival of DS children. The data showed that the presence of additional anomalies significantly influenced survival status. Unfortunately, information on the cause of death was unavailable to the study [33].

Morris et al. (2014) aimed to quantify the prevalence of congenital anomalies (diagnostic coding: ICD9/BPA or ICD10/BPA) in babies born with DS using data from the European Surveillance of Congenital Anomalies (EUROCAT) Central Register. The study population consisted of 27 registries in 18 countries, covering seven million births from 2000 to 2010. The final study included 14,109 cases with DS, comprising 6738 live births, 306 fetal deaths, and 7065 TOPFAs. Among 7044 live births and fetal deaths from 20 weeks of gestation, 2.3% had a severe CAKUT phenotype. However, the study did not compare these results with those of a control population. Unfortunately, CAKUT phenotypes were not specifically reported in TOPFAs. The study found that urinary anomalies were more common in males, with renal dysplasia being nine times more likely in males than females (OR = 8.00; 95% CI: 1.42–45.1). Limitations of the study included the fact that some registries did not provide information on whether a postmortem examination was performed, and several registries had a high proportion of missing information regarding postmortem examinations. Overall, 15.0% (14.2–15.8%) of births with DS had a non-cardiac congenital anomaly, while 16.8% (14.8–19.1%) of TOPFAs with a reported postmortem examination had a diagnosed non-cardiac congenital anomaly, which was significantly higher than in live births and fetal deaths. These findings highlight the prevalence and sex-specific differences in congenital anomalies among DS cases while also acknowledging the limitations in data reporting and postmortem examination information [27].

In 2018, Safdar et al. reported a high prevalence of CAKUT in a population of 241 patients with DS, finding that 21% of these patients had renal issues, making it the third most common congenital anomaly in this group. The renal abnormalities detected through imaging included hydronephrosis, VUR, obstruction, ectopic kidneys, and other conditions. The population size was small. On the other hand, only 139 patients underwent screening by ultrasound, which could have led to asymptomatic CAKUT cases remaining undetected. This highlights the need for comprehensive screening to better understand the full scope of renal abnormalities in DS patients. The outcomes for patients with renal involvement varied: 9.8% developed CKD, 15.7% died due to various causes, and the majority, 74.5%, showed no progression of the renal disease. This study did not include TOPFAs, miscarriages, or stillbirths. Also, it did not include any controls [35].

A recent meta-analysis by Rossetti et al. (2024) identified a 6.3% prevalence of CAKUT anomalies and a 50% prevalence of lower urinary tract dysfunction among individuals with DS. Their extensive literature search produced three case-control studies by Torfs and Christianson, Cleves et al., and Kallen et al., which examined the prevalence of CAKUT in around 18,000 individuals with DS and a control group exceeding 13 million subjects. Studies lacking controls and those with fewer than 10 subjects with DS were excluded. Their analysis showed that individuals with DS have a pooled relative risk of 5.49 for CAKUT compared to the general population. The most frequent malformations in DS were urethral anomalies (hypospadias and epispadias), obstructive malformations, a dilated urinary tract system, and kidney hypodysplasia. This meta-analysis underscores the higher incidence of various CAKUT defects, lower urinary tract dysfunctions, and slightly reduced kidney size in people with DS compared to the general population [24].

**Section summary:** Children with DS face an elevated risk of CAKUT, with prevalence estimates ranging from 0.22% to 21.16% across studies (median 2.88%). This wide range is largely due to methodological differences, including varying sample sizes, inclusion or exclusion of stillbirths and TOPFAs, and differences in postnatal surveillance duration. Multiple studies also report an increased risk of obstructive CAKUT defects among children with DS. To improve consistency across research, there is a critical need for standardized CAKUT phenotype classifications. Additionally, most birth defect registries are limited by diagnosing conditions within the first 1 to 2 years of life, underscoring the need for further studies with extended follow-up.

## 4. CAKUT Phenotypes in Down Syndrome

CAKUT classifications are generally organized according to the anatomical region affected within the urinary tract and the nature of each anomaly. In this review, we categorize these defects and related conditions into four primary groups: those involving the kidneys, ureters, bladder, and urethra. The following sections provide an overview of CAKUT phenotypes reported in DS, with a focus on specific anomalies within each category, as well as related conditions and processes (Table 3).

### 4.1. Kidney Anomalies

**Figure 1:**
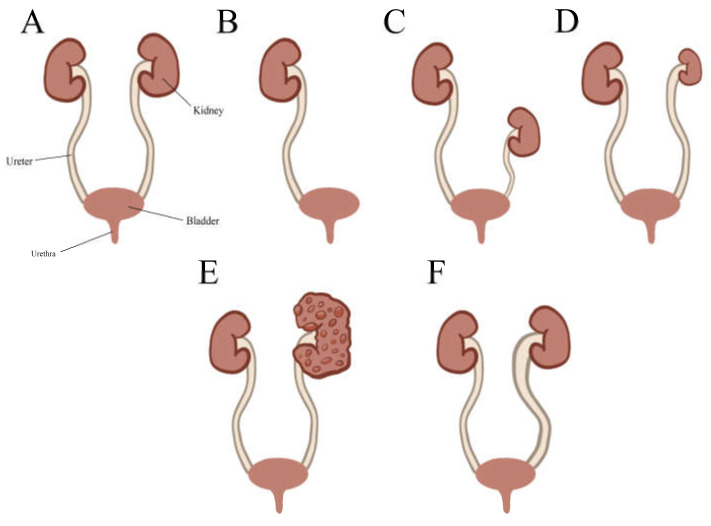
Schematic representation of various disorders of kidney development: (**A**) A normally developed urinary system consists of two kidneys (with the left kidney typically positioned slightly higher than the right), two ureters connecting to the bladder, and the urethra. Common urinary system disorders present in patients with Down syndrome include (**B**) unilateral kidney agenesis, (**C**) ectopic kidney, (**D**) kidney hypoplasia, (**E**) polycystic kidney disease, and (F) primary hydroureter.

Macroscopically, congenital kidney malformations are characterized by changes in the size, shape, position, or number of kidneys, while microscopically, they are defined by a lack of nephrons and/or abnormal histological structure. These anomalies can affect one or both kidneys and range from mild to severe, impacting kidney function and overall health [58] (Figure 1).

#### 4.1.1. Renal Agenesis

Renal agenesis is a congenital condition characterized by the absence of one or both kidneys (Figure 1B). On ultrasound, this is typically indicated by an absent bladder due to a lack of filling, missing renal tissue, and the absence of detectable blood flow in the renal arteries [22,58].

In cases of unilateral renal agenesis, patients are born with only one kidney, which compensates by increasing its filtration from birth. This hyperfiltration can lead to long-term issues such as adult-onset renal dysfunction and hypertension. Common symptoms include hyperuricemia, hematuria, and proteinuria. On the other hand, bilateral renal agenesis is a life-limiting condition. The lack of both kidneys causes severe anhydramnios (absence of amniotic fluid), often resulting in fetal death or neonatal pulmonary hypoplasia [22,58]. Epidemiological studies report that the prevalence of renal agenesis in the general population ranges from 0.00096% to 1.59%. In a meta-analysis involving over 15 million cases, the pooled prevalence of renal agenesis was found to be 0.03% (95% CI: 0.03–0.04%) [22].

Renal agenesis in DS was first reported by Berg et al. in 1960, based on autopsies of an infant and a two-year-old child [7]. The first population-based study to include any CAKUT phenotype was a multinational cohort study that examined the occurrence of 23 major malformations, including renal agenesis/dysgenesis in DS infants and stillbirths. Kallen et al. reported a prevalence of 0.04%, but they did not find an increased risk of kidney agenesis/dysgenesis compared to the general population. The study’s grouping of renal agenesis and dysgenesis without specifying differences may have impacted their results, as dysgenesis includes a range of kidney malformations with varying degrees of function, unlike complete absence in agenesis [30].

In their population-based study, Torfs and Christianson focused on anomalies less prone to misclassification, such as renal agenesis. Their study did not find an increased risk of renal agenesis in DS cases, with a prevalence of 0.03%. The authors suggested that by excluding stillbirths and TOPFAs, their study may have underestimated the prevalence of agenesis, as severe defects could lead to miscarriage or abortion [31]. A population-based study by Cleves et al. found a 0.03% prevalence of renal agenesis and reported no significant difference between infants with and without DS. The authors recognized several limitations, including the lack of information on whether karyotyping was performed to confirm DS and the exclusion of data on stillbirths and TOPFAs [32]. Kupferman et al. conducted a retrospective cohort study on CAKUT occurrence in a DS population compared to a control group. The prevalence of renal agenesis in DS children was 0.23% (0.04% in the control population) with a significantly increased risk (OR: 5.4 [95% CI: 2.8–10.4]). This finding is significant because it suggests a higher prevalence of renal agenesis in the DS population than previously reported, indicating potential underestimation in earlier studies [19]. Morris et al. reported four cases of bilateral renal agenesis, including Potter syndrome, in their population-based study of 7044 live births and fetal deaths with DS, resulting in a prevalence of 0.06%. However, the exclusion of unilateral renal agenesis likely underestimates the overall prevalence of renal agenesis in their findings [27].

**Section summary:** Although rare, renal agenesis can profoundly impact the quality of life. Prevalence rates in individuals with DS vary across studies, with some reporting no increased prevalence compared to the general population, while others found a significantly higher risk (OR: 5.4). Variability in study methodologies, including exclusion of stillbirths and TOPFAs and grouping of agenesis with other malformations, may have led to underestimations of its true prevalence in DS.

#### 4.1.2. Renal Hypoplasia

Kidney size or volume and length are significant indicators of its function. Also, it is a cardinal item in urinary imaging and evaluation of the total renal functions. Bilateral reduction of renal size is imperatively associated with chronic renal impairment, especially with glomerulonephritis and other systemic parenchymal medical disorders [59,60]. The length and size of the kidney correlate and are usually expressed relative to the whole-body anthropometric measures [58] (Figure 1D). 

The definition of hypoplastic kidneys has significantly evolved since the mid-20th century from being normal in structure but small due to a reduced number of papillae to a more refined understanding that includes functional and histological criteria [58]. Today, hypoplastic kidneys are defined as abnormally small kidneys, with kidney volume below two standard deviations of that of age-matched normal individuals or a combined kidney volume of less than half of what is normal for the patient’s age. Simple renal hypoplasia maintains normal corticomedullary differentiation and a reduced number of nephrons, while oligomeganephronic hypoplasia is characterized by marked compensatory growth of the reduced number of nephrons, glomerular hypertrophy, and tubular enlargement. Oligomeganephronic hypoplasia predisposes individuals to the development of focal segmental glomerulosclerosis (FSGS) due to adaptive responses to the reduced number of functioning nephrons [61]. A reduced kidney size in DS was demonstrated in two controlled studies and is supported by uncontrolled investigations. 

In 1967, Naeye conducted an autopsy study on the topic of abnormalities in prenatal growth in newborn infants with trisomies due to subnormal gestational size in most of these infants. This study presumably included 21 DS infants and reported a decrease in kidney mass by 31.1% (*p* < 0.001) by comparison with control infants of the same gestational age and weight. This was the first study that documented relatively small kidneys as a consistent abnormality in DS. It was also noted to be specific to DS, unlike cardiac abnormalities, which are present in other trisomies. A major limitation of this study was that no chromosomal studies were available for these infants, resulting in their classification as DS on the basis of phenotypic characteristics. Consequently, it is possible that a portion of these infants might not have had Trisomy 21 [8]. In their 1991 study, Ariel et al. defined renal hypoplasia as a reduction of at least one-third of the combined renal weight while maintaining structurally normal renal tissue as a prerequisite. Under these conditions, they found renal hypoplasia in 21.4% of DS cases and a reduction in combined kidney weight of a mean of 14.4% compared to the expected renal weight based on crown-heel length. Notably, the number of generations of glomeruli in these kidneys fell within the normal range, indicating normal nephrogenesis and highlighting an intriguing aspect of renal development in DS. They suggested that insufficient postnatal tubular growth was responsible for the notably smaller kidney due to tubular lengthening being the main factor of renal growth in the final phase of kidney development [10].

Recently, Postolache et al. (2022) performed a retrospective cohort study, which was the first to systematically compare the kidneys of 49 DS children with age- and sex-matched controls with the help of ultrasonography. They noted significant differences in kidney length (7.56 ± 1.2 vs. 8.27 ± 1.13 cm; *p* < 0.0001) and volume (52.2 ± 25.1 vs. 69.8 ± 29.2 cm^3^; *p* < 0.001) as well as kidney thickness at the hilum level (3.60 ± 0.66 vs. 4.20 ± 0.78; *p* < 0.001) even after normalization for body surface area (BSA). The data were normalized due to the fact that children with DS are known to be shorter and have a higher body BMI than corresponding controls [4].

Hypoplastic kidneys were first associated with DS newborns in the autopsy study by Berg et al., which described two cases of hypoplastic kidneys, one of which also had a hypoplastic bladder (ages 4 days and 1.5 months, respectively) [7]. The population-based study by Cleves et al. examined the prevalence of renal agenesis and hypoplasia among newborns with DS and concluded that there was no heightened prevalence [32]. Ebert et al. ultrasonically detected kidneys that were half the normal size in 2 out of 11 DS patients (18.2%) with bladder dysfunction [15].

**Section summary:** Several studies on individuals with DS have reported reductions in kidney mass, with one study noting a 14.4% and the other a 31.1% decrease in kidney size [8,10]. Additionally, a recent ultrasound study revealed significantly reduced kidney length (*p* < 0.0001) and volume (*p* < 0.001) [4]. These findings lead us to conclude that kidney size is reduced in individuals with DS, which suggests an increased tendency to develop CKD.

#### 4.1.3. Renal Ectopia

Renal ectopia is a congenital condition where a kidney fails to reach its standard location in the upper retroperitoneum. Instead, the kidney may be located in the pelvis, iliac region, abdomen, thorax, or even on the opposite side of the body (crossed ectopia) (Figure 1C). Occurring in about 0.14% of infants on ultrasound screenings, half of the ectopic kidneys are hydronephrotic, with causes including obstruction, VUR, or abnormal rotation without obstruction [62,63]. The intrathoracic kidney is a rare form with the lowest frequency among all renal ectopias. Interestingly, there have been 3 cases of this rare condition out of a total of 12 (25%) reported cases of renal ectopia in DS patients, warranting further investigation into the matter (Table 3).

#### 4.1.4. Renal Fusion (Horseshoe Kidney)

The most frequent type of renal fusion anomaly is the horseshoe kidney, which typically involves two kidney masses joined at their lower poles by either a parenchymal or fibrous isthmus resembling a horseshoe. While an isolated horseshoe kidney is usually considered benign, individuals with this condition have a greater incidence of UPJO, nephrolithiasis (kidney stones), and VUR than the general population [64].

Horseshoe kidneys were one of the first CAKUT phenotypes to be documented in DS by Berg et al. (1 in 141 DS cases, 0.7%) in their autopsy study [7]. Subsequent reports of this anomaly in DS have been limited. However, the population-based study by Torfs and Christianson included horseshoe kidneys as one of the anomalies analyzed in a DS population due to their lower susceptibility to misclassification compared to other CAKUT phenotypes. Their findings showed a significant association between DS and horseshoe kidneys (risk ratio 11.7, 0.001 < *p* < 0.05) with a prevalence of 0.07% [31]. This is much lower than the prevalence in other chromosomal disorders such as Turner syndrome (14% to 20%) and Edwards syndrome, where it reaches approximately 67% (attributed to a narrow pelvis) [65]. Further research is required to clarify the relationship between renal fusion anomalies and DS.

#### 4.1.5. Renal Cystic Disease

Renal cystic diseases that are evident in DS can be divided into three categories: glomerulocystic disease, simple renal cysts, and renal dysplasia with cysts [66]. Of the three, glomerulocystic disease has been reported in a far greater number of publications (Table 3) (Figure 1E). We will go through them and other literature related to renal cystic disease in DS succinctly in the following paragraphs.

Glomerulocystic kidney disease (GCKD) is a histologic description formerly known as glomerular microcysts that involves cyst formation in the glomerulus and dilatation of Bowman’s space and is the most commonly found cystic disease in DS kidneys. The appearance of the kidneys on ultrasound is echogenic, but the cysts are not always evident. Hereditary GCKD has typically been associated with small renal volumes such as those found in DS [4,66].

Simple cysts on ultrasound are characterized by sharp borders, clear sound wave transmission, and no internal echoes. These cysts average about 1 cm in size. The most common cause is hypertension, believed to result from hypertrophy due to localized ischemia or obstruction. Simple cysts are typically asymptomatic and do not impact renal function unless they are very large [66].

GCKD found mainly in the subcapsular region was described by Ariel et al. in 23.7% of autopsies of live-born DS cases. Since they were not mainly linked with obstructive defects, they concluded that they were an inherent morphologic characteristic in DS. Other than that, they found focal dilatation of tubules (8%) and simple cysts (6%) [10]. Lo et al. (1998) performed an autopsy study comparing 43 DS cases with 57 age-matched controls and confirmed that GCKD was more common in DS patients (65%) than in control subjects (25%), particularly in the first decade of life [12]. The findings of Naeye et al. revealed the mean diameter of glomeruli to be 16.2% larger compared to glomeruli in control infant kidneys, but this finding was not statistically significant [8]. Kupferman et al. identified a significantly increased risk of specific CAKUT phenotypes in their population-based study, including cystic dysplastic kidney, which had an OR of 4.5 compared to the control population [19].

More recently, Desogus et al. (2016) studied morphological changes in fetal DS kidneys and found that glomeruli in DS subjects were larger, which was attributed to increased Bowman’s space and had shape anomalies compared to those in normal subjects. Previous studies have shown that hyperfiltration and glomerular hypertrophy can lead to glomerular injury and nephron loss, contributing to CKD. An enlarged Bowman’s space, along with a reduced glomerular tuft, correlates with a lower number of functional glomeruli. This makes kidneys with many abnormal glomeruli vulnerable to significant nephron deficits and an increased risk of CKD in adulthood [67]. As these glomerular cysts grow, they cause tubular dilation and interfere with the development of additional nephrons, leading to abnormal kidney development in fetuses [68]. Therefore, glomerular cysts play a critical role in the progression to renal dysplasia, a primary cause of CKD in children with DS [58].

**Section summary**: The higher prevalence and distinct characteristics of renal cystic diseases in DS highlight a critical need for continued research to understand the pathogenesis and long-term implications of these renal abnormalities, as they play a significant role in the increased risk of CKD among individuals with DS.

#### 4.1.6. Hydronephrosis

Hydronephrosis is characterized as the dilation of the renal pelvis and calyces, which usually manifests as a consequence of impaired urine outflow distal to the renal pelvis. It is commonly found in conjunction with a dilated ureter and is then diagnosed under the joint term of hydroureteronephrosis [69].

While hydronephrosis itself is not a primary classification category of CAKUT, it is a common secondary feature associated with various primary CAKUT phenotypes, including UPJO, ureterocele, megaureter, PUV, VUR, and others. As a result, one study found that hydronephrosis was the most frequently reported CAKUT defect in non-DS newborns, accounting for 31.79% of all CAKUT cases in this population [20,69].

As for DS, according to available literature, hydronephrosis is by far the most frequently reported kidney malformation (Table 3). In the population-based study by Rankin et al., hydronephrosis was the only phenotype specified from the CAKUT spectrum, and their findings indicated a prevalence of 0.6% [33]. Kupferman et al. identified a significantly increased risk of specific CAKUT phenotypes, including hydronephrosis, which had an OR of 8.7 compared to the control population [19]. Hydronephrosis is easily detected in the first trimester during the routine exam of fetal anatomy, which could explain its high incidence in DS as well as non-DS infants. 

Studies have shown that the presence of bilateral hydronephrosis could be a valuable soft marker for aneuploidies during gestation. Dagklis et al. (2008) found that the prevalence of hydronephrosis was higher in DS in both the first and second trimesters than in chromosomally typical fetuses and concluded that including hydronephrosis as a risk factor could help improve the accuracy of screening for DS [70]. As a key indicator of underlying CAKUT, hydronephrosis necessitates careful evaluation and timely management, as early detection can be crucial for improving outcomes in affected newborns.

**Section summary:** Hydronephrosis, a common CAKUT feature, is the most frequently detected kidney malformation in DS. One study showed an increased risk in DS (OR: 8.7), and its prenatal detection may aid aneuploidy screening. Early diagnosis is crucial for better outcomes.

#### 4.1.7. Renal Pyelectasis

Pyelectasis, also known as pelviectasis, is identified by fluid accumulation within the renal pelvis. While both this condition and hydronephrosis involve dilation of the renal pelvis, pyelectasis is a milder form that may not indicate serious issues. In contrast, hydronephrosis involves significant dilation of both the calyces and pelvis, is more severe, and typically requires medical intervention [71].

According to currently available literature, pyelectasis is the least frequent kidney anomaly, with only four cases reported in two separate studies (Table 3). During gestation, pyelectasis is characterized by an enlargement of the renal pelvis, with a diameter exceeding 4–6 mm in the second trimester or 7–10 mm in the third trimester. Although often transient and clinically insignificant after birth, pyelectasis has been linked to an increased risk of aneuploidy, particularly DS. The incidence of aneuploidy in fetuses with pyelectasis ranges from 0.3% to 0.9%, with rates for DS reported between 10% and 25% [71,72,73,74,75]. In a 2012 systematic review, Orzechowski et al. analyzed 10 studies on fetuses with isolated pyelectasis in the second trimester and its association with DS. The review found that isolated fetal pyelectasis significantly increased the odds of DS, with a pooled positive likelihood ratio of 2.78 (95% CI, 1.75–4.43). However, the pooled negative likelihood ratio was 0.99 (95% CI, 0.98–1.00), suggesting that the absence of isolated pyelectasis does not reduce the likelihood of DS [76]. 

**Section summary:** As a soft marker for DS, pyelectasis is linked to a higher likelihood of aneuploidy, particularly when accompanied by other congenital anomalies or sonographic markers. Therefore, it should be carefully considered in prenatal screenings.

#### 4.1.8. Acquired Glomerulopathies

Although not classified as CAKUT phenotypes, acquired glomerulopathies are kidney disorders affecting the glomeruli that develop later in life, often as a consequence of certain CAKUT anomalies. CAKUT can predispose patients to these conditions through persistent urinary tract obstruction, infections, or high blood pressure. When CAKUT impairs baseline kidney function, the development of acquired glomerulopathies can further increase the risk of CKD progression, creating a cumulative impact on kidney health [58]. A wide spectrum of glomerulopathies have been reported in DS through small studies and sporadic case reports (Table 4). Given their association with CAKUT, we will briefly discuss them in the following paragraphs to address the continuum of kidney disease in DS individuals and highlight the increased vulnerability of their renal systems.

To our knowledge, the first to report acquired glomerulopathies in DS was a small case series by Gupta et al. (1991) documenting membranoproliferative glomerulonephritis in 4 patients with DS and progressive renal disease, with no apparent underlying etiology. All patients advanced to chronic renal failure and ultimately passed away, but those who received treatment prior to renal insufficiency experienced notable benefits [38].

Subsequent case reports described numerous glomerular lesions such as focal segmental glomerulosclerosis (FSGS), IgA glomerulonephritis, IgG glomerulonephritis, membranous glomerulonephritis, immunotactoid glomerulopathy, pauci-immune crescentic glomerulonephritis, Lupus nephritis, Anti-neutrophilic cytoplasmic antibody (ANCA)-associated glomerulonephritis, and membranoproliferative glomerulonephritis (Table 4).

Lo et al. (1998) performed an autopsy study focusing on glomerular diseases in DS patients and found several acquired lesions, including FSGS, minimal change disease (MCD), and membranous glomerulonephritis. However, these conditions had similar frequencies in DS and controls [12]. Later, Malaga et al. also stated that no specific glomerulopathy was associated with DS [13].

More recently, Said et al. examined kidney biopsies from 17 DS patients and found a wide array of glomerular diseases in their patient group (ages 6–45 years). Among them were already reported conditions (Table 4), but also one novel disease, lupus nephritis. They tracked 16 of the 17 subjects for an average of 47 months. Six progressed to ESRD, four died while on dialysis, one survived on dialysis, and one underwent a renal transplant at age 13, maintaining normal allograft function for 9 years [78].

Individuals with DS have a higher incidence of autoimmune disorders, which may contribute to the development of acquired glomerulopathies [88]. Many forms of glomerulonephritis, including lupus nephritis, IgA nephropathy, anti-neutrophilic cytoplasmic antibody (ANCA)-associated glomerulonephritis and membranous glomerulonephritis are believed to stem from immune system dysfunction, highlighting the need for further investigation into the potential link between DS-related immune dysregulation and renal disease [89].

**Section summary:** The relationship between DS and acquired glomerular lesions remains unclear due to the lack of large epidemiologic or observational studies. The largest study to date found no association between the two, despite 11 types of lesions being described in DS [12]. Further research is needed to determine the incidence of these lesions in DS patients. 

#### 4.1.9. Morphologic Changes of the Kidney

The nephron is the functional unit of the kidney, and its number determines kidney health throughout life. Unlike most other organs, the course of nephron formation begins in the 10th week and concludes by the 36th week of gestation, with most of them developing during the last trimester. Their number can range from 200,000 to 2 million units per kidney and is positively correlated with birth weight [58]. 

Chromosomal Trisomy is associated with altered cell-growth parameters, including slower growth and increased apoptosis. Children with DS exhibit a significantly higher rate of prematurity (gestational ages of 32 to 36 weeks) compared to non-DS neonates (21.1% vs. 6.3%) and a higher incidence of fetal growth restriction [26,90,91]. Additionally, DS infants born at full term (GA 40 weeks) consistently have lower mean birth weights, ranging between the 10th and 50th percentiles, which may result from prematurity or fetal growth restriction [91]. Since low birth weight is associated with a reduced nephron number, children with DS are likely to have fewer nephrons, increasing their susceptibility to CKD in adulthood [23]. In support of this, Naeye et al. found a 55% decrease in the number of glomeruli in DS infants compared to controls, indicating a lower nephron count [8].

Ariel et al. found immature glomeruli deep in the cortex in 15% of DS autopsies, indicating the expansion of the nephrogenic zone, which would suggest delayed renal maturation and may result in a reduced number of nephrons [10,67].

In a case report, a patient with DS and oligonephronia but no clinical symptoms or abnormal ultrasound findings underwent a renal biopsy, which revealed reduced glomeruli, mild tubular dilatation, and abnormal podocytes, indicating immature kidney development and confirmed oligonephronia as the cause of renal impairment [92]. One case report diagnosed a DS infant with renal tubular dysgenesis, a rare and severe congenital disorder characterized by a significant reduction to complete lack of proximal convoluted tubules (PCT), which leads to impaired kidney function, while the glomeruli and distal convoluted tubules develop normally. As a result of this condition, the infant developed severe fetal hydrops and died of respiratory failure soon after birth [93].

**Section summary:** The morphological changes observed in the kidneys of individuals with DS, including reduced nephron number, immature glomeruli, and other renal abnormalities, highlight the increased risk for kidney dysfunction and CKD in adulthood.

### 4.2. Ureter Anomalies

According to the literature, the ureter seems to be the least defect-afflicted organ of the urinary tract in DS. Among reported anomalies, we can find ureteral dilatation (hydroureter, megaureter), uretocele, ureteral stenosis, and duplication of the ureter and renal pelvis (Table 3).

#### 4.2.1. Ureteral Dilatation

Hydroureter, or in extreme cases megaureter, is the most common ureter anomaly to be found in patients with DS and is formed following the obstruction of urine outflow, which dilates, bends, elongates, and causes the affected ureter to become tortuous (Figure 1F). The condition is usually associated with other CAKUT anomalies, mostly hydronephrosis under the joint term hydroureteronephrosis. Similar to hydronephrosis, it is a frequent secondary finding and can be used to identify associated anomalies and to determine the location of obstructions in the genitourinary tract. Timely diagnosis and treatment of both conditions are essential to avoid serious kidney dysfunction [94,95].

Hydroureters were first described in DS by Egli and Stalder, who found a prevalence of 3% in their small cohort [9]. Afterward, they were mentioned in several studies including multiple population-based studies (Table 3). Notably, Kupferman et al. identified a significantly increased risk of hydroureters in a DS population with an OR of 8.5 compared to the control population [19]. Given the frequent association between hydroureters and hydronephrosis, and the high incidence of hydronephrosis in DS, it is likely that hydroureters are significantly underreported in DS [94].

#### 4.2.2. Ureteral Stenosis

Ureteral stenosis is a condition characterized by the narrowing of the ureter, which can obstruct the normal flow of urine and potentially lead to various symptoms and complications. When the narrowing occurs at the junction where the renal pelvis meets the ureter, the condition is referred to as UPJO, which is the most common cause of ureteral obstruction. Less frequently, when the narrowing occurs at the junction where the ureter connects to the bladder, it is termed ureterovesical junction obstruction (UVJO) [96]. 

These conditions are often referred to by different names, complicating the establishment of a uniform and comparable classification across studies. For example, ureteral stenosis and ureteral stricture are frequently used interchangeably. Although ureteral stenosis might not be specifically reported in most population-based studies, it falls within the spectrum of obstructive uropathy, which is commonly categorized under urogenital defects and has been shown to have a strong association with DS [10,19,32,34,35]. This suggests that ureteral stenosis is likely significantly underreported.

Naeye et al. were the first to report UPJO, which they referred to as “stricture of the uretero-pelvic junction”, in 2 out of 21 (9.5%) autopsies of individuals with DS. Notably, this was the only CAKUT phenotype among the seven reported in their study that was exclusively observed in DS and not in other trisomies such as Trisomy 18 (Edwards syndrome), Trisomy 13 (Patau syndrome), Trisomy 14, and Trisomy 15 [8]. Since then, there have been no reported cases of UPJO in Trisomy 18 or Trisomy 13. Kupferman et al. identified one case of UPJO among 3832 children with DS, with an OR of 1.4 compared to controls, a result that is not significant [19]. Given that UPJO is the most common cause of prenatally diagnosed hydronephrosis, which is the most frequently reported form of CAKUT in DS, it is reasonable to conclude that UPJO is likely underreported in the context of DS because of the frequent lack of use of specific diagnostic labels [94].

**Section summary:** Although the literature may indicate that ureteral anomalies are less frequent in DS, conditions such as hydroureter and UPJO are likely significantly underreported, which could alter this perception.

### 4.3. Bladder Anomalies

Although CAKUT anomalies are relatively common, those primarily affecting the bladder are less frequent. While many bladder anomalies can be detected prenatally through ultrasound, some are only identified at birth or later based on clinical symptoms [64]. In patients with DS, bladder anomalies such as PUV, VUR, bladder exstrophy, hypertrophy, trabeculation, and bladder neck stenosis have been reported (Table 3).

#### 4.3.1. Posterior Urethral Valves (PUVs)

**Figure 2:**
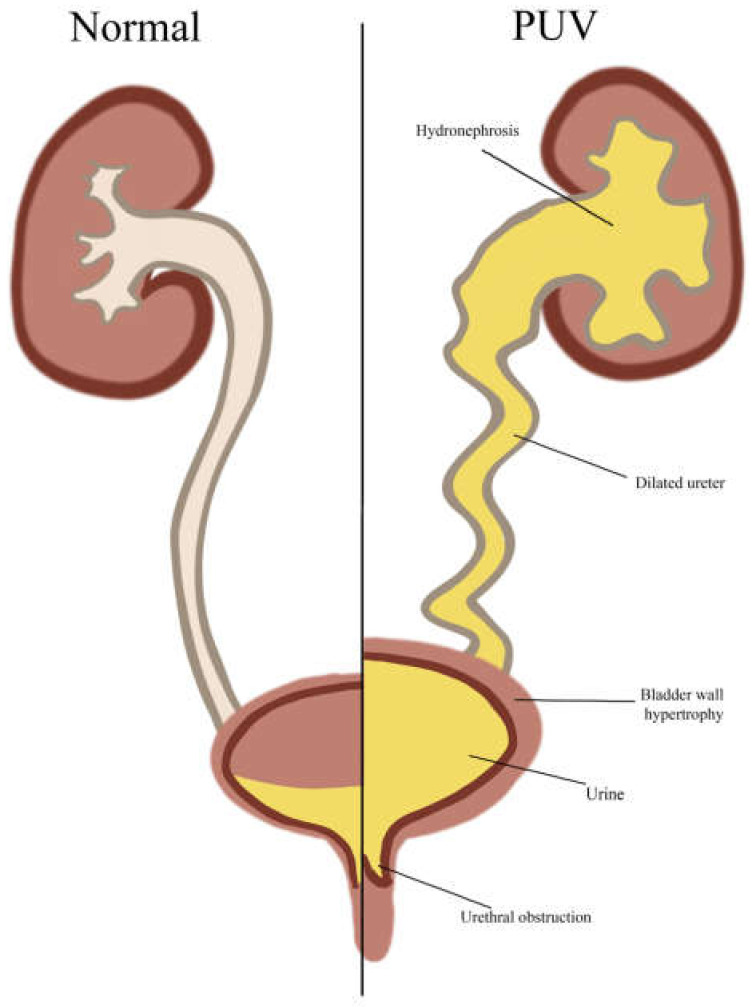
A side-by-side comparison of a normal urinary flow system and a system affected by hydronephrosis caused by posterior urethral valves (PUVs). In PUV, urine flow is obstructed by a valve-like tissue, leading to bladder wall hypertrophy, ureteral dilatation, and hydronephrosis in advanced stages.

PUVs are a serious condition and the most frequently found bladder malformations in DS, according to the current literature (Table 3). Intraluminal folds cause partial or complete obstruction of the posterior urethra, a part of the male urinary tract, thereby impeding urinary outflow exclusively in males (Figure 2). Infants with PUVs may develop CKD in 42.5% and progress to end-stage kidney disease in about 14.5% of cases. Indeed, 17% of children with kidney failure can be attributed to PUVs, with PUV-induced renal hypoplasia/dysplasia being one of the more common indications for pediatric renal transplants [97,98,99].

PUVs are most commonly detected during routine prenatal ultrasounds. Anomalies such as hydronephrosis, a distended bladder, and oligohydramnios can indicate urinary tract obstruction, prompting postnatal follow-up imaging and symptomatic evaluation. Some cases may not be diagnosed until later in infancy or early childhood when symptoms such as recurrent urinary tract infections, poor growth, or signs of kidney dysfunction become apparent [100,101]. 

PUVs were one of the first CAKUT phenotypes reported in a DS infant, first identified in a 1960 autopsy study by Berg et al. [7]. Since then, the association has been established in a few case reports and case series describing PUVs in DS (Table 3). Notably, in a population-based study by Kupferman et al., the odds of PUV occurrence in children with DS were found to be seven times higher than in the general population [19]. PUVs also fall into the category of obstructive defects and could account for a large number of the widely but vaguely reported obstructive defects in population-based studies. This would indicate that PUVs are severely underreported in DS, as is the case with other such defects.

Recently, Xiang et al. presented the largest known series of DS patients with PUVs. Their study included 18 DS patients with PUVs from their database along with 14 DS patients from various reviewed case series, totaling 32 patients with DS [19,52,72,102,103,104,105,106]. The authors found that, contrary to assumptions, the renal function, rates of progression to renal failure, the likelihood of achieving continence, and transplantation rates of DS patients with PUV were similar to those without DS. Furthermore, their study indicated a 3–4% risk of PUVs in patients with DS [54].

Narasimhan et al. encountered six cases of DS in a cohort of 230 patients with PUVs (2.6%), four of which had bilateral hydronephrosis, all with delayed diagnosis (age of presentation was between 1.5 and 7 years old). The babies had symptoms of urinary incontinence that were voiced by parents to the treating physicians but initially dismissed, which they attributed to the lack of attention to the association of DS with PUVs [48].

Since a conventional ultrasound may miss PUVs, voiding cystourethrography should be considered for boys with recurrent urinary tract infections or significant postnatal ultrasound findings [107]. With a higher incidence of PUVs in patients with DS, physicians must have a higher index of suspicion for PUV when patients with DS present with voiding dysfunction.

**Section summary:** PUVs are a common bladder malformation in males with DS, which significantly increases the risk of CKD and ESRD. Early detection and intervention are crucial to prevent renal failure. The occurrence of PUVs in children with DS is seven times higher than in the general population, with a 3–4% risk in DS patients. Despite the high incidence, renal function outcomes in DS patients with PUVs are similar to those without DS. Physicians should suspect PUVs in DS patients with voiding dysfunction to ensure timely treatment.

#### 4.3.2. Lower Urinary Tract Dysfunction (LUTD)

LUTD can be categorized into two main types: storage disorders and emptying disorders of the bladder. Bladder dysfunction is associated with intellectual disability, and children with DS typically take twice as long as their peers to achieve toilet training. Additionally, incontinence is common in DS children who have already been toilet trained, which could be linked to lower urinary tract dysfunction, among other factors [108,109,110,111].

The incidence and etiology of neurogenic or non-neurogenic (functional) storage or emptying disorders of the bladder in DS are only seldom reported. If unrecognized and untreated, bladder dysfunction can lead to secondary damage to the upper urinary tract and even to ESRD.

On this topic, Ebert et al. (2008) retrospectively analyzed 24 pediatric DS patients with urological problems and found that 11 patients had bladder storage and/or bladder emptying disorders (45.8%) by investigating abnormal micturition patterns, urinary incontinence, and urinary tract infections, 27.3% of which were neurogenic disorders and 72.7% were functional. All but one of the children were treated conservatively with behavioral therapy measures, consistent stool regulation, low-dose anticholinergics, and, if necessary, alpha blockers. Secondary complications, primarily VUR, were noted in 36.4% of subjects, and 27.3% had secondary hydronephrosis with megaureters. Due to severe functional impairment as a result of bladder dysfunction, three kidneys had to be removed. These findings indicate that a lack of initial screening and consistent long-term urological care can lead to serious upper urinary tract damage and unnecessary surgeries [15]. 

Although rare, one study suggested that male DS patients are more likely to develop non-neurogenic neurogenic bladder (NNB), a functional bladder disorder that mimics neurogenic bladder without a neurological basis, compared to those without DS [112,113]. Similar findings were observed in female DS patients in two case reports [50,114]. Notably, Kim et al. described a female DS patient who developed ESRD from untreated NNB, emphasizing the impact of delayed urological care in DS patients due to intellectual disability [50].

A recent meta-analysis by Rossetti et al. reviewed three studies on lower urinary tract dysfunction in DS, involving 169 DS patients and 172 controls. They found a 50.4% prevalence of bladder dysfunction and a nearly three times higher risk of bladder dysfunctions in individuals with DS as compared to healthy controls, though the studies showed high statistical heterogeneity (I^2^ = 81%) likely due to different diagnostic criteria [108,110,115].

Renal failure is a common outcome in patients with bladder storage or emptying disorders, though irreversible renal damage generally occurs only when the diagnosis is delayed, which is often the case in DS [50,116]. Clinicians should be attentive to symptoms such as incontinence, nocturia, frequent daytime urination, or weak urine stream in children with DS. Observing any of these signs should prompt an ultrasound of the urinary tract to evaluate for abnormalities [98,101]. 

**Section summary:** Early detection of secondary complications from bladder dysfunction is crucial to prevent progressive damage to the upper urinary tract and chronic renal failure. Because intellectual disabilities can make it challenging to identify voiding symptoms in DS patients, increased vigilance is required. Given the high prevalence of bladder dysfunction in this population, functional diagnostics should be conducted whenever bladder disorders are suspected.

### 4.4. Urethra Anomalies

#### Hypospadias and Epispadias

Hypospadias is a congenital condition where the opening of the urethra is located on the underside of the penis instead of at the tip and stems from incomplete development of the urethra and deficiency of the corpus spongiosum, while in epispadias the opening of the urethra is located on the upper surface of the penis in males or in an abnormal position on the urethra in females [18,117,118].

In the context of DS, the incidence of hypospadias is notably higher compared to the general population. A study by Lang et al. reported a hypospadias rate of 12% in males with DS, which was 40 times higher than in non-DS males [18]. Other studies have also highlighted elevated rates of hypospadias in DS patients. For instance, Stoll et al. reported a hypospadias incidence of 0.55% among DS cases over 26 years, compared to 0.18% in the general population, which translates to an OR of approximately 3.06 [34]. This means that hypospadias is about three times more likely to occur in individuals with DS than in those without. Torfs and Christianson found a risk ratio of 5.3 for hypospadias and epispadias in DS [31]. Cleves et al. reported an odds ratio of 2.1 (95% CI: 1.7–2.6) for these conditions, while Kupferman et al. calculated an odds ratio of 2 [19,32]. Morris et al. documented hypospadias in 24 out of 3905 DS males (0.61%) [27] (Table 3). Most recently, a meta-analysis by Rosetti et al. identified urethral anomalies, including hypospadias and epispadias, as the most frequent CAKUT malformations in DS [24]. On the other hand, one population-based study by Kallen et al. on 5581 infants with DS found hypospadias rates similar to those in the general population [30] (Table 3).

**Section summary:** Hypospadias is significantly more common in individuals with DS than in the general population. Studies report notably higher rates, with Lang et al. finding a 12% incidence in DS males, up to 40 times higher than in non-DS males [18]. Other studies also indicate increased odds ratios, confirming hypospadias as one of the most frequent urinary tract malformations associated with DS. 

### 4.5. Other

#### Obstructive Defects

Obstructive uropathy is characterized by impaired urine flow due to various congenital or acquired anomalies, which can potentially damage the kidneys. This condition may affect any part of the urinary tract, including the ureters, bladder, and urethra, and is commonly associated with anatomical abnormalities such as UPJO, PUVs, and urethral strictures. The severity and location of the obstruction determine the symptoms, which can range from abdominal pain and hematuria to urinary tract infections and, in severe cases, ESRD. Early detection through prenatal ultrasound or imaging techniques is crucial, as it can reveal signs like hydronephrosis, bladder distension, or other anomalies that warrant further investigation. In children, intrinsic causes such as urethral valves, strictures, and junctional stenosis are more common. If left untreated, these obstructions can lead to renal dysplasia, scarring, and irreversible kidney damage. Increased pressure in the urinary system due to obstruction disrupts kidney function, potentially causing chronic renal impairment [5,28,58]. 

Unspecified obstructive uropathy is the most reported CAKUT category in the DS population (Table 3). Despite its significance, reports of obstructive uropathy in DS lack detailed descriptions, highlighting the need for greater awareness and consistent diagnostic terminology to prevent long-term renal complications. For more information on specific obstructive defects in DS, please refer to earlier sections.

## 5. Kidney Function in Children with DS

Renal function assessment in children with DS has attracted significant attention in recent years due to the increasing recognition of kidney involvement in this population. Recently, Pautonnier et al. have shown that serum creatinine levels in French children with DS are higher than in the general pediatric population [119]. These findings, consistent with those of Nishino et al., highlight the importance of considering potential ethnic and genetic differences when evaluating kidney function, as variations in creatinine measurements are influenced by factors such as ethnicity and muscle mass [120,121].

Studies by Mariño-Ramírez et al. have further underscored the relevance of ethnic differences in kidney function biomarkers. Their investigation revealed that serum creatinine levels were significantly lower in East Asian children compared to their European counterparts, suggesting that creatinine reference values might need to be adjusted based on population-specific characteristics [121]. This notion is also supported by findings from Nishino et al., who demonstrated that children with DS had higher serum creatinine levels even in the absence of kidney, urological, neuromuscular, or congenital heart disease, further suggesting that DS itself may contribute to altered renal function [120].

In addition to the potential impact of ethnicity, other factors, such as the lower height and reduced muscle mass typical in children with DS, may also play a role in kidney function [120]. Creatinine, a marker commonly used to assess renal function, is typically influenced by muscle mass [122]. However, despite the reduced muscle mass seen in children with DS, serum creatinine levels tend to be elevated, which may indicate a degree of renal impairment [119]. This is further substantiated by studies showing that children with DS tend to have a lower estimated glomerular filtration rate (eGFR) compared to healthy children [4,13,120,121,123]. For example, Postolache et al. reported a mean eGFR of 94.3 ± 16.6 mL/min/1.73 m^2^ in a cohort of 49 Belgian children with DS, with 5 of the participants presenting with eGFR values below 75 mL/min/1.73 m^2^ [4]. In a Spanish cohort, Malaga et al. found that 4% of children with DS had eGFR values between 50 and 60 mL/min/1.73 m^2^ [13]. These findings suggest a higher prevalence of mild renal dysfunction in children with DS, even in the absence of overt kidney disease.

Further studies conducted in Japan have also confirmed lower eGFR in children with DS (124, 125, 125). Nishino et al. found that the mean eGFR in children with DS was 93.44 ± 17.14 mL/min/1.73 m^2^, which was approximately 20% lower than that of healthy Japanese children [124]. Similarly, Yamakawa et al. reported a median eGFR of 90 mL/min/1.73 m^2^ in a cohort of 108 Japanese children with DS [123]. These findings support the notion that renal function in DS children is systematically lower than in the general population, even when using various eGFR estimation formulas such as Schwartz 1976 [125], Schwartz 2009 [126], and Uemura’s equation [127].

The reduced eGFR in children with DS is particularly concerning, given the potential for long-term kidney dysfunction. As renal function assessment in DS can be complicated by factors such as altered growth patterns, delayed puberty, and differences in body composition (e.g., lower lean mass), some researchers have questioned the accuracy of using general population-based formulas for eGFR estimation in DS children [119,123,124] or even healthy children [128]. Specifically, children with DS may present with a different distribution of body fat and lean mass, particularly in males, which could result in an underestimation of renal function if standard formulas are applied [124].

Despite these challenges, serum creatinine remains a commonly used marker for detecting kidney dysfunction in clinical practice. However, there are some limitations to this approach. Serum cystatin C, another marker of renal function, may not be reliable in children with DS due to its dependence on thyroid function, which is often dysregulated in this population [129,130].

Therefore, while systematic monitoring of serum creatinine in children with DS is not universally mandated, it is advisable to monitor kidney function regularly in at-risk children, especially those with known cardiac or renal anomalies. In light of these findings, regular follow-up of renal function in children with DS is essential. As proposed by Ranchin et al., annual measurement of serum creatinine should be considered as part of routine health checks for children with DS [111]. Referral to a pediatric nephrologist may be necessary if creatinine levels exceed the 97.5th percentile of the standard pediatric curves, or if eGFR falls below threshold values indicating mild kidney dysfunction [119,123] (Table 5).

## 6. Genetic Factors Contributing to CAKUT in Individuals with DS

Understanding the molecular pathways and genetic factors that increase the susceptibility of individuals with DS to CAKUT is a complex challenge. The link between an extra chromosome 21 and the DS phenotype was discovered in 1959 and marked a milestone in genetic medicine [23]. In 2000, the sequencing of the long arm of chromosome 21 (HSA21) was completed and published, and with that, research and understanding of the molecular mechanisms underlying DS advanced significantly [132]. Today, DS is recognized as a gene-dosage disorder, with partial or complete Trisomy 21 as its underlying genomic cause [3].

The current GENCODE/ENSEMBL database (release 46) catalogs 233 protein-coding genes, 423 non-coding genes, and 188 pseudogenes on HSA21, though nearly half remain unannotated due to repetitive elements [90]. Despite recent advances, studying the effects of triplicated protein-coding and non-coding genes and their downstream consequences remains difficult, particularly in animal models [3]. Two primary hypotheses have been proposed to explain DS phenotypes. The first is the HSA21 gene-dosage effect, where overexpression of HSA21 genes disrupts biological processes. The second is developmental instability, suggesting that the extra HSA21 causes widespread disruption of gene expression, leading to biological imbalance [133,134]. Both mechanisms are thought to contribute to DS-related phenotypes [3].

Certain genes in the DS critical region have been linked to CAKUT. Having certain genes in this region in triplicate may increase the risk of developing CAKUT. One of the most studied is the dosage-sensitive DYRK1A (dual-specificity tyrosine-(Y)-phosphorylation-regulated kinase 1 A), which encodes a highly conserved kinase, is known for its role in neurodevelopment and is mapped to 21q22.13. An increase in the dosage of DYRK1A has been linked to several DS phenotypes, such as intellectual disability [135], congenital heart defects [136], craniofacial dysmorphology [137], and retinal disorders [138]. However, one study expanded the phenotype of DYRK1A-related intellectual disability syndrome (caused by mutations or deletions in DYRK1A) to include CAKUT. Blackburn et al. investigated CAKUT in 19 patients with de novo putative loss-of-function variants in DYRK1A. Patients exhibited features of DYRK1A-related intellectual disability syndrome, which include autism, microcephaly, developmental delay, seizures, and others. CAKUT phenotypes were present in 73% of patients, including unilateral renal agenesis, mild pelviectasis, and hypospadias. Additionally, they provided supporting evidence using Xenopus embryos to demonstrate DYRK1A’s role in various kidney developmental processes. Highlighting the importance of DYRK1A’s kinase domain in nephrogenesis, dyrk1a knockdown resulted in abnormal tubule formation or, in some cases, complete loss of the kidney [139]. This research indicates that the dosage sensitivity of DYRK1A could contribute to the renal phenotypes observed in individuals with DS.

UBASH3A (Ubiquitin Associated and SH3 Domain Containing A), a gene located in the DS critical region of HSA21 (21q22.3), appears to play a significant role in kidney development by influencing cell fate decisions and key signaling pathways necessary for embryonic growth [140,141]. Although the precise mechanisms remain unclear, UBASH3A is thought to be crucial for proper kidney formation and morphogenesis [140]. This gene shows high expression in early kidney structures like tubules and glomeruli, particularly during prenatal stages of rapid kidney growth. Through its involvement in ubiquitin-mediated signaling, particularly its interaction with proteins like Syk, UBASH3A likely influences kidney patterning and cell proliferation [140,141,142,143]. 

On the topic of epigenetics, Yuen et al. conducted a comparison of normal and trisomic kidney tissues, focusing on DNA methylation patterns. The study analyzed DNA methylation across roughly 1000 CpG sites in regulatory regions of nearly 800 genes, using tissue samples from second-trimester elective terminations of eight normal and five DS fetuses. The findings indicated no significant differences in DNA methylation between normal and DS kidney tissues [144].

One challenge in identifying the exact genetic and other effects of Trisomy 21 is that studies in humans are limited to cases that survive to birth. This means that only genetic variations compatible with life are considered when studying DS-related conditions [3]. Investigating how genetic variation contributes to the diverse phenotypic characteristics of CAKUT in DS is crucial. By sequencing the genomes of thousands of individuals with DS and linking these genetic differences to their specific phenotypic traits, researchers could begin to explore these relationships. 

**Section summary:** The genetic mechanisms underlying CAKUT in DS remain unclear. Trisomy 21 leads to overexpression of genes like *DYRK1A* and *UBASH3A*, both implicated in kidney development. *DYRK1A* mutations have been linked to CAKUT phenotypes, with functional studies in *Xenopus* embryos supporting its role in nephrogenesis. *UBASH3A* is thought to influence kidney morphogenesis through ubiquitin-mediated signaling. While a study found no significant DNA methylation differences between normal and trisomic kidney tissues, further research is needed to fully understand the genetic and epigenetic contributions to CAKUT in DS.

## 7. Methods for Diagnosing CAKUT in Individuals with DS and the Importance of Early Detection and Screening Protocols

Early detection and diagnosis of CAKUT in individuals with DS are crucial for improving outcomes and preventing long-term complications [111]. In developed countries, routine prenatal screening for DS is a standard part of antenatal care. This screening typically includes maternal serum analyte measurements and ultrasound, which help identify pregnancies at high risk for DS. In the 1980s, the focus was on α-fetoprotein levels, and more recently, the inclusion of additional markers such as β-human chorionic gonadotropin and pregnancy-associated plasma protein A has improved screening accuracy [25]. With the advent of cell-free DNA testing in 2011, sequencing of maternal serum became a reliable, non-invasive method for detecting DS, providing valuable information for clinicians and parents. However, while these methods can indicate risk, they do not provide a definitive diagnosis, and further diagnostic testing, such as amniocentesis or chorionic villus sampling, is recommended when needed [3].

In addition to genetic screening, prenatal ultrasonography is routinely used to detect potential anatomical anomalies in the fetus. Although no specific fetal anatomical findings are diagnostic of DS, ultrasound can reveal signs of abnormalities in the kidneys or urinary tract. For instance, hydronephrosis and cystic structures in the renal system may suggest underlying CAKUT [40,130]. Early identification of these issues can lead to timely interventions, which may improve developmental outcomes. Post-mortem MRI has been shown to enhance the accuracy of prenatal diagnoses by providing detailed imaging of organs that may be difficult to assess with conventional ultrasound [40].

Despite the importance of early detection, many cases of renal anomalies in DS are still underdiagnosed. Research by Narasimhan et al. highlighted the challenges of identifying renal issues in DS patients, such as delayed diagnosis of PUVs, a condition that may go unnoticed due to a lack of awareness among healthcare providers. Routine screening through ultrasound and micturating cystourethrogram (MCU) is recommended for all DS infants to ensure that urological anomalies are promptly identified and treated [48].

Given the increased prevalence of renal and urological anomalies in individuals with DS, experts recommend systematic screening starting in infancy. Regular monitoring of renal function, including urinalysis and ultrasound, should continue throughout life to detect and manage CAKUT early. Early intervention can prevent progression to CKD and improve the overall quality of life for individuals with DS.

## 8. Medical and Surgical Management Options for CAKUT in Individuals with DS

The quality of life for individuals with DS is often impacted by physical health issues, particularly those involving the kidneys. Timely screening and early interventions are critical in addressing CAKUT. Recognizing and managing these anomalies early can help mitigate their adverse effects on development. While kidney transplantation and dialysis are options for patients with ESRD associated with DS, they present unique challenges. For instance, peritoneal dialysis (PD) in DS patients can be complicated by an increased risk of peritonitis, often due to difficulties in maintaining personal hygiene and treatment compliance. As a result, renal transplantation is typically considered the preferred treatment option [52].

There is limited research on the outcomes of renal replacement therapy (RRT) in individuals with DS, but some reports suggest that both hemodialysis and PD can be performed successfully, despite complications such as noncompliance [39,45,51,52,55,145]. However, patients with DS often face additional barriers, including cognitive impairments and co-morbid conditions, which can complicate their care. 

Renal transplantation in individuals with DS and renal failure are seldom reported, primarily within the pediatric population [47,51,145,146,147]. One early case in 1995 described a successful living donor kidney transplant from a mother to her 14-year-old daughter with DS [147]. Additionally, between 1987 and 1995, a study in the U.S. followed 14 DS children who received kidney transplants, with 10 patients on dialysis at the time and 4 undergoing preemptive transplants. By 1998, nine of the transplants were still functioning, while two failed due to acute rejection. The researchers concluded that renal transplantation is a viable option for renal replacement therapy in DS patients [85]. In contrast, only two adult DS patients who received kidney transplants have been reported, both of whom had diabetic nephropathy [145].

**Section summary:** The decision to pursue renal replacement therapy in DS patients is complex and often influenced by factors such as family support, social environment, and the patient’s ability to follow medical protocols. These challenges make it difficult to standardize treatment for DS patients with CAKUT. Additionally, many infants with DS may have other congenital defects that require specialized care, making it important for healthcare providers to be aware of the potential for multiple medical needs and to plan treatments accordingly [32]. While advancements in healthcare have improved the life expectancy and quality of life for individuals with DS, more research is necessary to develop targeted medical and surgical strategies that address both the kidney-related and broader health challenges faced by these individuals [52,145].

## 9. Conclusions

In conclusion, individuals with DS are at an elevated risk for CAKUT, which can lead to significant kidney dysfunction and CKD in adulthood. Despite the high prevalence (ranging from 0.22% to 21.16% across studies) of these conditions, there is a lack of standardized diagnostic criteria and extended follow-up studies. 

Our findings indicate that hydronephrosis is the most common renal anomaly in DS patients, followed by renal hypoplasia and glomerulocystic disease. Ureteral dilation is the most frequent ureteral abnormality, while PUVs commonly affect the bladder. Additionally, hypospadias appears very frequently as a urethral anomaly. Multiple studies also report a high number of unspecified obstructive CAKUT defects along with an increased risk among children with DS. Several studies have reported reductions in kidney mass, length, and volume, suggesting an increased susceptibility to CKD.

Children with DS and CAKUT may face a distinct progression of CKD compared to non-DS children with CAKUT. While CAKUT is the leading cause of pediatric CKD, DS-specific factors such as reduced kidney size, lower nephron number, glomerular abnormalities, and a higher prevalence of bladder dysfunction (e.g., PUVs) may contribute to greater CKD risk and progression. Despite these concerns, data directly comparing CKD progression between DS and non-DS children with CAKUT are lacking. Further research is needed to clarify these differences and guide tailored management strategies.

Overall, published reports suggest a broad spectrum of CAKUT phenotypes in DS, even when considering terminology inconsistencies that may contribute to underreporting. Early detection through systematic screening, starting in infancy, is essential for timely intervention and CKD prevention. Furthermore, research into the genetic factors of CAKUT in DS is critical to improving diagnostic accuracy and treatment strategies. While genetic data linking DS and CAKUT remains limited, the available evidence suggests a role for specific genes like *DYRK1A* and *UBASH3A*. Understanding how these and other dosage-sensitive genes on HSA21 contribute to kidney development could provide new insights into the pathogenesis of CAKUT in DS and help guide future genetic and therapeutic research.

However, several limitations in current studies must be acknowledged. The exclusion of stillbirths, miscarriages, and TOPFAs may underestimate the true prevalence of CAKUT in DS. Additionally, heterogeneity in data collection methods, diagnostic criteria, and reporting practices complicate cross-study comparisons. The frequent misclassification of CAKUT phenotypes and reliance on early-life diagnoses further contribute to underreporting, particularly for anomalies that manifest later in life. Standardized classification systems and extended follow-up studies are needed to improve research accuracy and clinical management.

While these limitations suggest that the prevalence of CAKUT in DS is likely underappreciated, similar challenges exist in estimating CAKUT prevalence in the general population. CAKUT is often an incidental finding later in life, especially in mild cases, and its true prevalence in the broader population remains difficult to determine. This underscores the broader issue of estimating CAKUT prevalence in humans and emphasizes the need for improved screening methods across all populations.

Given the complexity of managing these issues alongside other congenital defects in DS patients, healthcare providers should adopt a proactive, lifelong approach to care, including regular renal monitoring throughout life. Future studies should focus on refining diagnostic methods, implementing long-term follow-up protocols, and improving imaging techniques to enhance CAKUT detection. Increased awareness and further research are necessary to develop tailored medical strategies that address the unique health challenges of individuals with DS.

## Figures and Tables

**Table 1:** Autopsy and small studies that have reported CAKUT phenotypes in DS, excluding case reports.

Authors, Reference no.	Year of Publication	Country	Study Design	No. of Subjects with DS	No. of Subjects with CAKUT and DS	% of CAKUT	Age Range
Berg et al. [7]	1960	UK	autopsy	141	5	3.55	0–27 y, SB
Naeye [8]	1967	USA	autopsy	21	3	14.29	36–40 gw
Egli and Stalder [9]	1973	Switzerland	autopsy	103	7	6.80	(−14)
Ariel et al. [10]	1991	USA	autopsy	124	NA	NA	16 gw–25 y
Subrahmanyam and Mehta [11]	1995	USA	retrospective chart review	54	1	1.85	2 m–24 y
Lo et al. [12]	1998	Hong Kong, USA	case study and retrospective autopsy review	45	29	64.44	NA
Malaga et al. [13]	2004	Spain	cross-sectional study	69	2	2.90	1–24 y
Hicks et al. [14]	2007	UK	retrospective chart review and cross-sectional study	7	7	100.00	0–9 y
Ebert et al. [15]	2008	Germany	retrospective chart review	24	24	NA	3 m–27 y
Solomon et al. [16]	2010	USA	case study	2	2	NA	6 w; 18 y
Jain et al. [17]	2014	India	cross-sectional study	40	NA	0.00	0–18 y
Postolache et al. [4]	2022	Belgium	retrospective cohort study	49	7	14.29	0–18 y
Lang et al. [18]	1987	USA	cross-sectional study	91	5	5.49	NA

gw—gestation weeks, m—months, NA—not applicable, SB—stillbirths, y—years.

**Table 2:** Population-based studies that have reported on the prevalence of CAKUT in DS.

Authors, Reference no.	Year of Publication	Country	Study Years	No. of Subjects with DS	No. of CAKUT	% of CAKUT	No. of Controls	Age Range
Stoll et al. [29]	1990	France	1979–1987	139	4	2.88	NA	20 gw–9 d
Kallen et al. [30]	1996	France, Italy, Sweeden	1976–1993	5581	12	0.22	#	NA
Torfs and Christianson [31]	1998	USA (California)	1983–1993	2894	56	1.94	2,490,437	0–1 y
Cleves et al. [32]	2007	USA	1993–2002	11372	991	8.71	7,884,209	newborns
Kupferman et al. [19]	2009	USA (New York State)	1992–2004	3832	123	3.21	3,411,833	0–2 y
Rankin et al. [33]	2012	UK	1985–2008	1115	11	0.99	NA	SB, miscarriages, TOPFAs, 0–12 y (1985–2001), 0–16 y (2001–2003)
Morris et al. [27]	2014	18 EU countries	2000–2010	7025 ^†^	159	2.26	NA	20 gw–1 y, SB
Stoll et al. [34]	2015	France	1979–2008	728	28	3.85	401,804	0–2 y, SB, TOPFAs
Safdar et al. [35]	2018	Saudi Arabia	2005–2016	241	51	21.16	NA	1 m–57 y

^†^—total number was 14,109, which included 7065 TOPFAs, but these were excluded from analysis because CAKUT was not reported in TOPFAs. #—Defect rates of the control population were calculated from ICBDMS report (36). NA—not applicable, SB—stillbirths, TOPFAs—Terminations of Pregnancy for Fetal Anomaly, gw—gestational weeks, d—days, y—years.

**Table 3:** Studies linked to CAKUT in DS.

CAKUT	Authors and Year	Study Cases	Total
			Reported
Kidney Anomalies			
Renal agenesis	Berg et al., 1960 [7]	2 of 141 DS autopsies	33 *
	Insunza et al., 1993 [37]	1 DS autopsy case of Potter syndrome	
	Kallen et al., 1996 [30]	2 agenesis/dysgenesis of 5581 DS infants	
	Torfs and Christianson, 1998 [31]	1 of 2894 DS infants	
	Cleves et al., 2007 [32]	14 agenesis/hypoplasia of 43,463 DS infants	
	Kupferman et al., 2009 [19]	9 of 3832 DS infants	
	Morris et al., 2014 [27]	4 of 7044 DS live births and fetal deaths	
Renal hypoplasia	Berg et al., 1960 [7]	2 of 141 DS autopsies	42 *
	Ariel et al., 1991 [10]	18 of 124 DS autopsies	
	Gupta et al., 1991 [38]	2 cases	
	Lo et al., 1998 [12]	1 of 45 cases	
	Malaga et al., 2004 [13]	1 of 69 DS cases	
	Cleves et al., 2007 [32]	14 agenesis/hypoplasia of 11,372 DS newborns	
	Ebert et al., 2008 [15]	2 out of 11 DS cases	
	Kute et al., 2013 [39]	1 case	
	Jain, 2014 [17]	NA	
	Staicu et al., 2015 [40]	1 case	
Renal ectopia	Boronat et al., 1982 [41]	1 case of intrathoracic kidney	12
	Stein, 1999 [42]	1 case of intrathoracic kidney	
	Malaga et al., 2004 [13]	1 of 69 DS cases	
	Navarro et al., 2005 [43]	1 case of intrathoracic kidney	
	Al-Manjomi et al., 2005 [44]	1 case of intrathoracic kidney	
	Kupferman et al., 2009 [19]	1 of 3832 DS infants	
	Kosmadakis et al., 2013 [45]	1 case	
	Safdar et al., 2018 [35]	5 of 241 DS cases	
Renal fusion (Horseshoe kidney)	Berg et al., 1960 [7]	1 of 141 DS autopsies	5
	Naeye, 1967 [8]	1 of 21 DS autopsies	
	Stoll et al., 1990 [29]	1 of 139 DS fetuses/newborns	
	Torfs and Christianson, 1998 [31]	2 of 2894 DS infants	
Renal dysplasia	Morris et al., 2014 [27]	11 of 7025 DS fetuses/infants/SB	12
	Anriquez et al., 2023 [46]	1 case	
Renal cystic disease			
Glomerulocystic disease	Ariel et al., 1991 [10]	23 of 124 DS autopsies	41
	Lo et al., 1998 [12]	28 of 45 cases	
Simple renal cysts	Ariel et al., 1991 [10]	7 of 124 DS autopsies	8
	Staicu et al., 2015 [40]	1 case	
Renal dysplasia with cysts	Ariel et al., 1991 [10]	4 of 124 DS autopsies	8
	Webb et al., 1993 [47]	1 case	
	Kupferman et al., 2009 [19]	3 of 3832 DS infants	
Hydronephrosis	Naeye, 1967 [8]	1 of 21 DS autopsies	~203
	Egli and Stalder, 1973 [9]	1 of 103 DS autopsies	
	Ariel et al., 1991 [10]	3 of 124 DS autopsies	
	Kupferman et al., 1995 [19]	2 cases	
	Narashiman et al., 2005 [48]	4 out of 6 DS cases with PUV	
	Hicks et al., 2007 [14]	7 of 7 DS children	
	Ebert et al., 2008 [15]	7 of 24 DS cases	
	Weijerman et al., 2008 [49]	2 of 176 DS infants	
	Kupferman et al., 2009 [19]	69 of 3832 DS infants	
	Rankin et al., 2012 [33]	12 of 1115 DS SBs, miscarriages, TOPFAs	
	Jain, 2014 [17]	8 of 40 DS children	
	Morris et al., 2014 [27]	66 of 7044 DS live births and fetal deaths	
	Staicu et al., 2015 [40]	1 case	
	Safdar et al., 2018 [35]	18 of 241 DS cases	
	Postolache et al., 2022 [4]	2 of 49 DS children	
Pyelectasis	Malaga et al., 2004 [13]	1 of 69 DS cases	4
	Postolache et al., 2022 [4]	3 of 49 DS children	
Ureter Anomalies			
Ureteral dilatation (hydroureter, megaureter)	Egli and Stalder, 1973 [9]	3 of 103 DS autopsies	28 *
	Stoll et al., 1990 [29]	1 of 139 DS fetuses/newborns	
	Ariel et al., 1991 [10]	6 of 124 DS autopsies	
	Hicks et al., 2007 [14]	5 of 7 DS children	
	Ebert et al., 2008 [15]	7 of 24 DS cases	
	Kupferman et al., 2009 [19]	5 of 3832 DS infants	
	Jain, 2014 [17]	NA	
	Kim et al., 2019 [50]	1 case	
Uretocele	Ariel et al., 1991 [10]	1 of 124 DS autopsies	
Ureteral stenosis	Egli and Stalder, 1973 [9]	2 of 103 DS autopsies	10 *
	Ariel et al., 1991 [10]	2 of 124 DS autopsies	
	Subrahmanyam and Mehta, 1995 [11]	1 of 54 DS cases	
	Jain, 2014 [17]	NA	
	Ebert et al., 2008 [15]	2 cases of UPJO of 24 DS cases	
	Kupferman et al., 2009 [19]	1 case of UPJO of 3832 DS infants	
	Naeye, 1967 [8]	2 cases of UPJO of 21 DS autopsies	
Duplication of the ureter and renal pelvis	Ariel et al., 1991 [10]	1 of 124 DS autopsies	
Bladder Anomalies			
PUV	Berg et al., 1960 [7]	1 of 141 DS autopsies	41 *
	Webb et al., 1993 [47]	2 cases	
	Kupferman et al., 1995 [51]	3 cases	
	Hausmann et al., 2002 [52]	1 case	
	Narashiman et al., 2005 [48]	6 cases	
	Ebert et al., 2008 [15]	2 of 24 DS cases	
	Kupferman et al., 2009 [51]	2 of 3832 DS infants	
	Morris et al., 2014 [27]	4 cases of PUV and/or prune belly of 7044 DS live births and fetal deaths	
	Lazarus et al., 2014 [53]	2 cases	
	Xiang et al., 2023 [54]	18 cases	
VUR	Stoll et al., 1990 [29]	1 of 139 DS fetuses/newborns	20 *
	Webb et al., 1993 [47]	2 cases	
	Kupferman et al., 1995 [51]	1 case	
	Narashiman et al., 2005 [48]	3 out of 6 DS cases with PUV	
	Hicks et al., 2007 [14]	3 of 7 DS children	
	Yavascan et al., 2008 [55]	1 case	
	Ebert et al., 2008 [15]	4 of 24 DS cases	
	Solomon et al., 2010 [16]	1 case	
	Jain, 2014 [17]	NA	
	Safdar et al., 2018 [35]	4 of 241 DS cases	
Bladder exstrophy	Stoll et al., 1990 [29]	1 of 139 DS fetuses/newborns	9 *
	Hausmann and Vandersteen, 1999 [56]	1 case	
	Reutter et al., 2006 [57]	1 case	
	Ebert et al., 2008 [15]	2 of 24 DS cases	
	Solomon et al., 2010 [16]	1 case	
	Morris et al., 2014 [27]	3 cases of bladder exstrophy and/or epispadias of 7044 DS live births and fetal deaths	
Bladder hypertrophy	Egli and Stalder, 1973 [9]	3 of 103 DS autopsies	6
	Ariel et al., 1991 [10]	1 of 124 DS autopsies	
	Malaga et al., 2004 [13]	1 of 69 DS cases	
	Anriquez et al., 2023 [46]	1 case of megabladder	
Trabeculated bladder	Ariel et al., 1991 [10]	3 of 124 DS autopsies	10
	Kupferman et al., 1995 [51]	2 cases	
	Hicks et al., 2007 [14]	5 of 7 DS children	
Bladder neck stenosis	Ariel et al., 1991 [10]	3 of 124 DS autopsies	3
Urethra Anomalies			
Urethral atresia	Solomon et al., 2010 [16]	1 case	1
Hypospadias/epispadias	Lang et al., 1987 [18]	5 of 77 DS males	467
	Kallen et al., 1996 [30]	10 of 5581 DS cases	
	Torfs and Christianson, 1998 [31]	36 of 2894 DS infants	
	Cleves et al., 2007 [32]	355 of 11,372 DS newborns	
	Ebert et al., 2008 [15]	2 of 24 DS cases	
	Kupferman et al., 2009 [19]	31 of 3832 DS infants	
	Morris et al., 2014 [27]	24 of 7044 DS live births and fetal deaths	
	Stoll et al., 2015 [34]	4 of 728 DS SBs/TOPFAs/infants	
Other			
Unspecified obstructive uropathy	Ariel et al., 1991 [10]	8 of 124 DS autopsies	665
	Cleves et al., 2007 [32]	638 of 11,372 DS newborns	
	Stoll et al., 2015 [34]	14 of 728 DS SBs/TOPFAs/infants	
	Safdar et al., 2018 [35]	4 of 241 DS cases	
Anterior urethral obstruction	Kupferman et al., 2009 [19]	1 of 3832 DS infants	

* Numbers might not be accurate due to undisclosed information in the original studies. CAKUT—Congenital Anomalies of the Kidney and Urinary Tract, DS—Down Syndrome, NA—not applicable, SB—stillbirth, TOPFA—Terminations of Pregnancy for Fetal Anomaly, UPJO—ureteropelvic junction obstruction, VUR—vesicoureteral reflux, PUV—posterior urethral valve.

**Table 4:** Studies that have reported acquired glomerulopathies in DS.

Acquired Glomerulopathies	Authors and Year	Study Cases	Total Reported
Membranoproliferative glomerulonephritis	Gupta et al., 1991 [38] Birk et al., 1996 [77] Said et al., 2012 [78] Alsultan et al., 2022 [79]	4 cases 1 case 2 of 17 biopsies 1 case	8
Renal amyloidosis	Ozkaya et al., 2005 [80]	1 case	1
Immunotactoid glomerulopathy	Takemura et al., 1993 [81]	1 case	1
Anti-neutrophilic cytoplasmic antibody (ANCA)-associated glomerulonephritis	Robson et al., 1995 [82] Haseyama et al., 1998 [83] Schwab et al., 1996 [84]	1 case 1 case 1 case	3
Focal segmental glomerulosclerosis (FSGS)	Lo et al., 1998 [12] Baqi et al., 1998 [85] Said et al., 2012 [78]	2 cases 2 cases 4 of 17 biopsies	8
Minimal change disease (MCD)	Lo et al., 1998 [12]	1 of 43 autopsies	1
IgA glomerulonephritis	Birk et al., 1996 [77] Said et al., 2012 [78]	1 case 5 of 17 biopsies	6
IgG glomerulonephritis	Assadi, 2004 [86]	1 case	1
Membranous glomerulonephritis	Lo et al., 1998 [12] Said et al., 2012 [78]	1 of 43 autopsies 1 of 17 biopsies	2
Pauci-immune crescentic glomerulonephritis	Birk et al., 1996 [77] Cherif et al., 2013 [87] Said et al., 2012 [78]	1 case 1 case 2 of 17 biopsies	4
Lupus nephritis	Said et al., 2012 [78]	1 of 17 biopsies	1

**Table 5:** Summary of studies on kidney function estimation in DS.

	Pautonnier et al. 2023 [119]	Nishino et al. 2021 [120]	Postolache et al. 2021 [4]	Nishino et al. 2020 [124]	Yamakawa et al. 2018 [123]	Malaga et al. 2005 ^¥^ [13]
	Retrospective 2004–2021	Retrospective 2003–2020	Retrospective cohort study 2017–2019	Retrospective 2003–2018	Retrospective 2002–2014	Cross-sectional
Number of children	279	568	49	379	108	69
Age, years Median	0–10	0.3–18	0–18 8.0 ± 4.2	2–18	2–18	1–24 9.7
Sex M F	162 117	336 232	30 19	224 155	59 49	37 32
Exclusion criteria	Yes ^£^	Yes ^©^	Yes ™	Yes ^®^	Yes ^∞^	No
Country	France	Japan	Belgium	Japan	Japan	Spain
Number of creatinine measurements	857	3765	ns	2421	ns	ns
Method	enzymatic	enzymatic	Jaffe	enzymatic	enzymatic	ns
Serum creatinine (μmol/L) Median	24.0–50.0	* 0.3–9 y 21.2–38.9 9–17 y Boys: 41.5–76.0 Girls: 39.8–62.8	* 44.2	ns	* 33.6	* 16.5 y M: 114.1 3.3 y M: 107.0
Serum creatinine (mg/dl) All children Boys: Girls:	0.27–0.57	0.3–9 y 0.24–0.44 9–17 y 0.47–0.86 0.45–0.71	0.5 ± 0.1	ns	0.38	16.5 y M:1.29 3.3 y M: 1.21
eGFR mL/min/1.73 m^2^ All children BoysGirls	ns	ns	94.3 ± 16.6	93.4 ± 17.14 90.86 ± 15.7 96.3 ± 18.21	90.0	16.5 y: 50.1 3.3 y: 56.3
Formula	ns	ns	Bedside Schwartz formula	Uemura	Uemura	Schwartz formula
Limitations	1. single-center design 2. relatively low number of children 3. not take sex into account 4. only French children were studied	1. single-center design 2. only Japanese children were studied	1. utilized a Jaffe creatinine assay, as opposed to an enzymatic assay, as used in the Bedside Schwartz eGFR formula 2. only Belgian children were studied	1. single-center design 2. included premature births and low-birth-weight infants 3. only Japanese children were studied	1. small number of subjects 2. the single-centerdesign 3. the short observation period 4. only Japanese children were studied	1. single-center design 2. only Spanish children were studied

^¥^ all ^¥^ patients with DS, not only children. ^£^ urinary or renal diseases (i.e., vesicoureteral reflux, renal insufficiency, renal dysplasia, or any renal anomaly detected by ultrasound, which was performed prenatally or postnatally in all children). ^©^ diseases affecting S–Cr (chronic glomerulonephritis (CGN)), congenital anomalies of the kidney and urinary tract (CAKUT) detected using abdominal ultrasonography, vesicoureteral reflux (VUR), infectious and inflammatory diseases, dehydration, muscle disease, neurological disease, malignant tumors, hypertension, severe CHD, liver or pancreatic diseases, and combinations of DS and other malformative syndromes. ™ age- and sex-matched controls were excluded if they had already known kidney and urinary tract anomalies. ^®^ congenital heart disease, congenital anomalies of the kidney or urinary tract detected via abdominal ultrasonography, chronic glomerulonephritis and vesicoureteral reflux, etc. ^∞^ urological malformation, infectious disease, inflammatory disease, dehydration, severe heart disorder with cyanosis or heart failure, uncontrolled thyroid disease, neurological disorder, and muscle abnormalities. ns not specified. * results converted from mg/mL using conversion factor 88.42 [131].

## References

[1] De Graaf G., Buckley F., Skotko B.G. (2021). Estimation of the number of people with Down syndrome in Europe. Eur. J. Hum. Genet..

[2] Gruhn J.R., Zielinska A.P., Shukla V., Blanshard R., Capalbo A., Cimadomo D., Nikiforov D., Chan A.C.H., Newnham L.J., Vogel I. (2019). Chromosome errors in human eggs shape natural fertility over reproductive lifespan. Science.

[3] Antonarakis S.E., Skotko B.G., Rafii M.S., Strydom A., Pape S.E., Bianchi D.W., Sherman S.L., Reeves R.H. (2020). Down syndrome. Nat. Rev. Dis. Primers.

[4] Postolache L., Parsa A., Simoni P., Boitsios G., Ismaili K., Schurmans T., Monier A., Casimir G., Albert A., Parsa C.F. (2022). Widespread kidney anomalies in children with Down syndrome. Pediatr. Nephrol..

[5] Chevalier R.L. (2023). CAKUT: A Pediatric and Evolutionary Perspective on the Leading Cause of CKD in Childhood. Pediatr. Rep..

[6] Down J.L. (1995). Observations on an ethnic classification of idiots. Ment. Retard..

[7] Berg J.M., Crome L., France N.E. (1960). Congenital cardiac malformations in mongolism. Br. Heart J..

[8] Naeye R.L. (1967). Prenatal organ and cellular growth with various chromosomal disorders. Biol. Neonatorum.

[9] Egli F., Stalder G. (1973). Malformations of kidney and urinary tract in common chromosomal aberrations. I. Clinical studies. Humangenetik.

[10] Ariel I., Wells T.R., Landing B.H., Singer D.B. (1991). The urinary system in Down syndrome: A study of 124 autopsy cases. Pediatr. Pathol..

[11] Subrahmanyam A.B., Mehta A.V. (1995). Renal anomalies in Down syndrome. Pediatr. Nephrol..

[12] Lo A., Brown H.G., Fivush B.A., Neu A.M., Racusen L.C. (1998). Renal disease in Down syndrome: Autopsy study with emphasis on glomerular lesions. Am. J. Kidney Dis..

[13] Málaga S., Pardo R., Málaga I., Orejas G., Fernández-Toral J. (2005). Renal involvement in Down syndrome. Pediatr. Nephrol..

[14] Hicks J.A., Carson C., Malone P.S.J. (2007). Is there an association between functional bladder outlet obstruction and Down’s syndrome?. J. Pediatr. Urol..

[15] Ebert A.K., Brookman-Amissah S., Rösch W.H. (2008). Urological manifestations of Down syndrome: Significance and long-term complications—Our own patient cohort with an overview. Urologe.

[16] Solomon B.D., Bous S.M., Bianconi S., Pineda-Alvarez D. (2010). Consideration of VACTERL association in patients with trisomy 21. Clin. Dysmorphol..

[17] Jain M., Singh A., Mantan M., Kapoor S. (2014). Evaluation of structural anomalies of kidney and urinary tract in children with Down syndrome. Indian. J. Pediatr..

[18] Lang D.J., Van Dyke D.C., Heide F., Lowe P.L. (1987). Hypospadias and urethral abnormalities in Down syndrome. Clin. Pediatr..

[19] Kupferman J.C., Druschel C.M., Kupchik G.S. (2009). Increased prevalence of renal and urinary tract anomalies in children with Down syndrome. Pediatrics.

[20] Sanna-Cherchi S., Westland R., Ghiggeri G.M., Gharavi A.G. (2018). Genetic basis of human congenital anomalies of the kidney and urinary tract. J. Clin. Investig..

[21] Sanna-Cherchi S., Kiryluk K., Burgess K.E., Bodria M., Sampson M.G., Hadley D., Nees S.N., Verbitsky M., Perry B.J., Sterken R. (2012). Copy-Number Disorders Are a Common Cause of Congenital Kidney Malformations. Am. J. Hum. Genet..

[22] Plutecki D., Kozioł T., Bonczar M., Ostrowski P., Skorupa A., Matejuk S., Walocha J., Pękala J., Musiał A., Pasternak A. (2023). Renal agenesis: A meta-analysis of its prevalence and clinical characteristics based on 15,641,184 patients. Nephrology.

[23] Lejeune J., Turpin R., Gautier M. (1959). Chromosomic diagnosis of mongolism. Arch. Fr. Pediatr..

[24] Rossetti C.M., Simonetti G.D., Bianchetti M.G., Lava S.A.G., Treglia G., Agostoni C., Milani G.P., de Winter J.P. (2024). Kidney and urogenital abnormalities in Down syndrome: A meta-analysis. Ital. J. Pediatr..

[25] Savva G.M., Morris J.K., Mutton D.E., Alberman E. (2006). Maternal Age-Specific Fetal Loss Rates in Down Syndrome Pregnancies. https://obgyn.onlinelibrary.wiley.com/doi/10.1002/pd.1443.

[26] Yao R., Contag S.A., Goetzinger K.R., Crimmins S.D., Kopelman J.N., Turan S., Turan O.M. (2020). The role of fetal growth restriction in the association between Down syndrome and perinatal mortality. J. Matern.-Fetal Neonatal Med..

[27] Morris J.K., Garne E., Wellesley D., Addor M.C., Arriola L., Barisic I., Beres J., Bianchi F., Budd J., Dias C.M. (2014). Major congenital anomalies in babies born with Down syndrome: A EUROCAT population-based registry study. Am. J. Med. Genet. Part A.

[28] Murugapoopathy V., Gupta I.R. (2020). A Primer on Congenital Anomalies of the Kidneys and Urinary Tracts (CAKUT). Clin. J. Am. Soc. Nephrol. CJASN.

[29] Stoll C., Alembik Y., Dott B., Roth M.P. (1990). Epidemiology of Down syndrome in 118,265 consecutive births. Am. J. Med. Genet..

[30] Källén B., Mastroiacovo P., Robert E. (1996). Major congenital malformations in Down syndrome. Am. J. Med. Genet..

[31] Torfs C.P., Christianson R.E. (1998). Anomalies in Down syndrome individuals in a large population-based registry. Am. J. Med. Genet..

[32] Cleves M.A., Hobbs C.A., Cleves P.A., Tilford J.M., Bird T.M., Robbins J.M. (2007). Congenital defects among liveborn infants with Down syndrome. Birth Defects Res. Part A—Clin. Mol. Teratol..

[33] Rankin J., Tennant P.W.G., Bythell M., Pearce M.S. (2012). Predictors of survival in children born with Down syndrome: A registry-based study. Pediatrics.

[34] Stoll C., Dott B., Alembik Y., Roth M.P. (2015). Associated congenital anomalies among cases with Down syndrome. Eur. J. Med. Genet..

[35] Safdar O., Albloushy R., Sait S., Almadani S., Ismail A. (2018). Incidence and outcome of renal anomalies in children with down syndrome. Australas. Med. J..

[36] Congenital Malformations Worldwide: A Report from the International Clearinghouse for Birth Defects Monitoring Systems | GHDx. https://ghdx.healthdata.org/record/congenital-malformations-worldwide-report-international-clearinghouse-birth-defects.

[37] Insunza A., González F., Guzmán E., Nielsen E., Gómez C., Castillo S., Valdés A. (1993). Potter syndrome caused by bilateral renal agenesis and duodenal atresia. Rev. Chil. Obstet. Ginecol..

[38] Gupta S.K., Venkataseshan V.S., Churg J. (1991). Mesangiocapillary glomerulonephritis in Down’s syndrome. Am. J. Nephrol..

[39] Kute V.B., Vanikar A.V., Shah P.R., Gumber M.R., Patel H.V., Engineer D.P., Thakkar U.G., Trivedi H.L. (2013). Down syndrome with end-stage renal disease. Indian. J. Clin. Biochem..

[40] Staicu A., Farcasanu A.S., Caracostea G., Turcu R.V.F., Simon S., Stamatian F. (2016). Contribution of post-mortem MRI to the evaluation of subtle renal anomalies in a first trimester foetus with Down syndrome. J. Obstet. Gynaecol..

[41] Boronat F., Pérez Bustamante I., Mayayo T., Gallego N., Romero C. (1982). Thoracic ectopic right kidney associated with Down syndrome and a serious heart abnormality. Actas Urol. Esp..

[42] Stein J.P., Kurzrock E.A., Freeman J.A., Esrig D., Ginsberg D.A., Grossfeld G.D., Hardy B.E. (1999). Right intrathoracic renal ectopia: A case report and review of the literature. Tech. Urol..

[43] Navarro A., Jiménez J., Ríos T., Mestanza F., Aguirre I., Urquizo R. (2005). Unusual cause of lung and renal disease in a baby with trisomy 21. Pediatr. Pulmonol..

[44] Al-Manjomi F.M., Al Mane K., Al-Nasser A. (2005). A three-year old girl with Down’s syndrome and an abnormal finding in the chest. Ann. Saudi Med..

[45] Kosmadakis G., Smirloglou D., Gobou A., Draganis T., Michail S. (2013). Hemodialysis treatment on an adult patient with Down syndrome associated with ectopic right kidney chronic obstructive nephropathy and secondary amyloidosis. Saudi J. Kidney Dis. Transpl..

[46] Anriquez D.A., Pussetto M.B., Seia F. (2023). Kidney trasplantation in a female infant with Down syndrome, Pseudo Prune Belly Syndrome, and a post-transplant lymphoproliferative disorder. First case report in Argentina. Rev. Nefrol. Dial. Traspl..

[47] Webb N., Hébert D., Arbus G. (1993). Renal replacement therapy in Down’s syndrome. Pediatr. Nephrol..

[48] Narasimhan K.L., Kaur B., Marwaha R.K. (2005). Posterior urethral valves in patients with Down syndrome. Indian. J. Pediatr..

[49] Weijerman M.E., van Furth A.M., Vonk Noordegraaf A., van Wouwe J.P., Broers C.J.M., Gemke R.J.B.J. (2008). Prevalence, neonatal characteristics, and first-year mortality of Down syndrome: A national study. J. Pediatr..

[50] Kim G.E., Sin D.S., Kim S.S., Lee C.-H., Cho N.-J., Lee E.Y. (2019). End-stage renal disease in a Down syndrome patient caused by delayed diagnosis of nonneurogenic bladder: A case report. Medicine.

[51] Kupferman J.C., Stewart C.L., Kaskel F.J., Katz S.P., Fine R.N. (1994). Chronic peritoneal dialysis in a child with Down syndrome. Pediatr. Nephrol..

[52] Hausmann M.J., Landau D. (2002). A Down syndrome patient treated by peritoneal dialysis. Nephron.

[53] Lazarus J., Theron A., Smit S. (2015). Posterior urethral valves and Down syndrome. Afr. J. Urol..

[54] Xiang A., Weaver J., Nadeem I., D’Souza N., Rickard M., Weiss D., Milford K., Woo L., Hannick J., Lorenzo A. (2023). Posterior urethral valves in patients with trisomy 21: Similar renal outcomes and rates of volitional voiding. J. Pediatr. Urol..

[55] Yavascan O., Kara O.D., Anil M., Bal A., Pehlivan O., Aksu N. (2008). Chronic peritoneal dialysis treatment in a pediatric patient with Down syndrome. Perit. Dial. Int..

[56] Husmann D.A., Vandersteen D.R., Gearhart J.P., Mathews R. (1999). Anatomy of Cloacal Exstrophy. The Exstrophy—Epispadias Complex: Research Concepts and Clinical Applications.

[57] Reutter H., Betz R.C., Ludwig M., Boemers T.M. (2006). MTHFR 677 TT genotype in a mother and her child with Down syndrome, atrioventricular canal and exstrophy of the bladder: Implications of a mutual genetic risk factor?. Eur. J. Pediatr..

[58] Potter E.L. (1972). Normal and Abnormal Development of the Kidney.

[59] El-Reshaid K., El-Reshaid W., Al-Bader D., Varro J., Madda J., Sallam H.T. (2017). Biopsy of small kidneys: A safe and a useful guide to potentially treatable kidney disease. Saudi J. Kidney Dis. Transpl..

[60] Matsell D.G., Cojocaru D., Matsell E.W., Eddy A.A. (2015). The impact of small kidneys. Pediatr. Nephrol..

[61] Paueksakon P., Fogo A.B. (2014). Autopsy Renal Pathology. Surg. Pathol. Clin..

[62] Rowe C.K., Merguerian P.A. (2024). Developmental Abnormalities of the Genitourinary System. Avery’s Diseases of the Newborn.

[63] Rosenblum N.D. (2018). Kidney Development. National Kidney Foundation’s Primer on Kidney Diseases.

[64] Devlieger R., Hindryckx A., Pandya P.P., Oepkes D., Sebire N.J., Wapner R.J. (2020). 33—Kidney and Urinary Tract Disorders. Fetal Medicine.

[65] Je B.-K., Kim H.K., Horn P.S. (2015). Incidence and Spectrum of Renal Complications and Extrarenal Diseases and Syndromes in 380 Children and Young Adults With Horseshoe Kidney. AJR Am. J. Roentgenol..

[66] Goksu S.Y., Leslie S.W., Khattar D. (2024). Renal Cystic Disease.

[67] Desogus M., Crobe A., Fraschini M., Ottonello G., Melania P., Faa G., Fanos V. (2016). Morphological changes in the kidney of fetuses with Down syndrome. J. Pediatr. Neonatal Individ. Med..

[68] Nagata M., Shibata S., Shu Y. (2002). Pathogenesis of dysplastic kidney associated with urinary tract obstruction in utero. Nephrol. Dial. Transplant..

[69] Patel K., Batura D. (2020). An overview of hydronephrosis in adults. Br. J. Hosp. Med..

[70] Dagklis T., Plasencia W., Maiz N., Duarte L., Nicolaides K.H. (2008). Choroid plexus cyst, intracardiac echogenic focus, hyperechogenic bowel and hydronephrosis in screening for trisomy 21 at 11 + 0 to 13 + 6 weeks. Ultrasound Obstet. Gynecol..

[71] Rao R., Platt L.D. (2016). Ultrasound screening: Status of markers and efficacy of screening for structural abnormalities. Semin. Perinatol..

[72] Kim D.-Y., Mickelson J.J., Helfand B.T., Maizels M., Kaplan W.E., Yerkes E.B. (2009). Fetal pyelectasis as predictor of decreased differential renal function. J. Urol..

[73] Coco C., Jeanty P. (2005). Isolated fetal pyelectasis and chromosomal abnormalities. Am. J. Obstet. Gynecol..

[74] Benacerraf B.R., Mandell J., Estroff J.A., Harlow B.L., Frigoletto F.D. (1990). Fetal pyelectasis: A possible association with Down syndrome. Obstet. Gynecol..

[75] Chudleigh P.M., Chitty L.S., Pembrey M., Campbell S. (2001). The association of aneuploidy and mild fetal pyelectasis in an unselected population: The results of a multicenter study. Ultrasound Obstet. Gynecol..

[76] Orzechowski K.M., Berghella V. (2013). Isolated fetal pyelectasis and the risk of Down syndrome: A meta-analysis. Ultrasound Obstet. Gynecol..

[77] Birk P.E., Burke B.A., Vernier R.L. (1996). Glomerulonephritis in children with Down syndrome. Pediatr. Nephrol..

[78] Said S.M., Cornell L.D., Sethi S., Fidler M.E., Al Masri O., Marple J., Nasr S.H. (2012). Acquired glomerular lesions in patients with Down syndrome. Hum. Pathol..

[79] Alsultan M.K., Bdeir Z.N., Obeid A., Alsamarrai O., Nabil Al Houri H., Hassan Q. (2022). Primary membranoproliferative glomerulonephritis in a child with down syndrome complicated with CVA: A case report. Ann. Med. Surg..

[80] Ozkaya O., Paksu M.S., Bek K., Yildiz L., Fişgin T., Gürmen N., Karagöz F. (2006). Renal amyloidosis due to pulmonary tuberculosis in a patient with Down syndrome. Eur. J. Pediatr..

[81] Takemura T., Yoshioka K., Akano N., Michihata I., Okada M., Maki S., Shigematsu H. (1993). Immunotactoid glomerulopathy in a child with Down syndrome. Pediatr. Nephrol..

[82] Robson W.L., Leung A.K., Woodman R.C., Trevenen C.L. (1995). Anti-neutrophil cytoplasmic antibody associated glomerulonephritis in a patient with Down’s syndrome. Pediatr. Nephrol..

[83] Haseyama T., Imai H., Komatsuda A., Hamai K., Ohtani H., Kibira S., Miura A.B. (1998). Proteinase-3-antineutrophil cytoplasmic antibody (PR3-ANCA) positive crescentic glomerulonephritis in a patient with Down’s syndrome and infectious endocarditis. Nephrol. Dial. Transplant..

[84] Schwab M., Böswald M., Ludwig K., Wittekind C., Waldherr R., Ruder H. (1996). A patient with Down’s syndrome and anti-neutrophilic cytoplasmic antibody-positive vasculitis. Pediatr. Nephrol..

[85] Baqi N., Tejani A., Sullivan E.K. (1998). Renal transplantation in Down syndrome: A report of the North American Pediatric Renal Transplant Cooperative Study. Pediatr. Transplant..

[86] Assadi F.K. (2004). IgG-associated mesangial glomerulonephritis in a patient with Down syndrome. Med. Sci. Monit..

[87] Cherif M., Hedri H., Ounissi M., Gergah T., Goucha R., Barbouch S., Abderrahim E., Maiz H.B., Kheder A. (2013). Pauci-immune crescentic glomerulonephritis in the Down’s syndrome. Saudi J. Kidney Dis. Transpl..

[88] Ferrari M., Stagi S. (2021). Autoimmunity and Genetic Syndromes: A Focus on Down Syndrome. Genes.

[89] Al-Aubodah T.-A., Piccirillo C.A., Trachtman H., Takano T. (2025). The autoimmune architecture of childhood idiopathic nephrotic syndrome. Kidney Int..

[90] Antonarakis S.E. (2017). Down syndrome and the complexity of genome dosage imbalance. Nat. Rev. Genet..

[91] Voigt M., Fusch C., Olbertz D., Hartmann K., Rochow N., Renken C., Schneider K. (2006). Analysis of the neonatal collective in the Federal Republic of Germany 12th report: Presentation of detailed percentiles for the body measurement of newborns. Geburtshilfe Frauenheilkd..

[92] Wada N., Miyazaki K., Enya T., Okada M., Sugimoto K. (2020). Renal impairment associated with oligonephronia in a patient with Down syndrome. Pediatr. Int..

[93] Jain V., Beneck D. (2003). Renal tubular dysgenesis in an hydropic fetus with trisomy 21: A case report with literature review. Pediatr. Dev. Pathol..

[94] Thotakura R., Anjum F. (2024). Hydronephrosis and Hydroureter.

[95] Osmundson S.S. (2021). Hydroureter. Am. J. Obstet. Gynecol..

[96] Vemulakonda V.M. (2021). Ureteropelvic junction obstruction: Diagnosis and management. Curr. Opin. Pediatr..

[97] Brownlee E., Wragg R., Robb A., Chandran H., Knight M., McCarthy L. (2019). Current epidemiology and antenatal presentation of posterior urethral valves: Outcome of BAPS CASS National Audit. J. Pediatr. Surg..

[98] Thakkar D., Deshpande A.V., Kennedy S.E. (2014). Epidemiology and demography of recently diagnosed cases of posterior urethral valves. Pediatr. Res..

[99] Tambo F.F.M., Tolefac P.N., Ngowe M.N., Minkande J.Z., Mbouche L., Guemkam G., Telelen N.A., Angwafo F.F., Sosso A.M. (2018). Posterior urethral valves: 10 years audit of epidemiologic, diagnostic and therapeutic aspects in Yaoundé gynaeco-obstetric and paediatric hospital. BMC Urol..

[100] Meneghesso D., Partigiani N.B., Spagnol R., Brazzale A.R., Morlacco A., Vidal E. (2023). Nadir creatinine as a predictor of renal outcomes in PUVs: A systematic review and meta-analysis. Front. Pediatr..

[101] Julie K., Chrystelle L., Cécile C., Justyna S., Petra Z., Mohammed D., Françoise M., Benjamin B., Angelique S., William M. (2013). Fetal Urinary Peptides to Predict Postnatal Outcome of Renal Disease in Fetuses with Posterior Urethral Valves (PUV). Sci. Transl. Med..

[102] Bielek J., Carvajal-Busslinger M.I., Bianchetti M.G. (1996). Posterior urethral valves in trisomy 21. Pediatr. Nephrol..

[103] Mondal K., Maheshwari A., Aneja S., Seth A. (2012). A case of Down syndrome with a posterior urethral valve. Indian. J. Nephrol..

[104] Danacıoğlu Y.O., Karaman M.İ., Çaşkurlu T., Sılay M.S. (2019). Congenital megalourethra and posterior urethral valve in a patient with Down syndrome. Turk. J. Urol..

[105] Garriboli M., Ibrahim S., Clothier J. (2021). Spontaneous bladder rupture secondary to posterior urethral valves in a boy with Down syndrome. BMJ Case Rep..

[106] Ahmed S. (1990). Vesico-ureteric reflux in Down’s syndrome: Poor prognosis. Aust. N. Z. J. Surg..

[107] Frimberger D., Mercado-Deane M.G., McKenna P.H., Austin J.C., Austin P.F., Cooper C.S., Greenfield S.P., Herndon C.D., Kolon T.F., MacNeily A.E. (2016). Establishing a Standard Protocol for the Voiding Cystourethrography. Pediatrics.

[108] Powers M.K., Brown E.T., Hogan R.M., Martin A.D., Ortenberg J., Roth C.C. (2015). Trends in Toilet Training and Voiding Habits among Children with Down Syndrome. J. Urol..

[109] Mrad F.C.d.C., de Figueiredo A.A., de Bessa J., Bastos Netto J.M. (2018). Prolonged toilet training in children with Down syndrome: A case–control study. J. Pediatr..

[110] Kızılay F., İrer B., Özalp Kızılay D., Şimşir A., Kalemci S., Şen V., Altay B., Çoğulu Ö. (2020). Evaluation of lower urinary tract symptoms in children with Down syndrome: A prospective, case-controlled cohort study. Neurourol. Urodyn..

[111] Ranchin B., Bidault V., Zekre F., DeMul A., Sanlaville D., Bacchetta J. (2024). Kidney and urological involvement in Down syndrome: Frequent, underestimated, but associated with impaired quality of life and risk of kidney failure. Pediatr. Nephrol..

[112] Handel L.N., Barqawi A., Checa G., Furness P.D., Koyle M.A. (2003). Males with Down’s syndrome and nonneurogenic neurogenic bladder. J. Urol..

[113] Groutz A., Blaivas J.G., Pies C., Sassone A.M. (2001). Learned voiding dysfunction (non-neurogenic, neurogenic bladder) among adults. Neurourol. Urodyn..

[114] Kai N., Seki N., Hirata A., Nakamuta S., Naito S. (2007). A female case with Down syndrome and non-neurogenic neurogenic bladder. Int. J. Urol..

[115] Kitamura A., Kondoh T., Noguchi M., Hatada T., Tohbu S., Mori K.-I., Matsuo M., Kunitsugu I., Kanetake H., Moriuchi H. (2014). Assessment of lower urinary tract function in children with Down syndrome. Pediatr. Int..

[116] Nijman R.J. (2001). Neurogenic and non-neurogenic bladder dysfunction. Curr. Opin. Urol..

[117] van der Horst H.J.R., de Wall L.L. (2017). Hypospadias, all there is to know. Eur. J. Pediatr..

[118] Baskin L.S., Ebbers M.B. (2006). Hypospadias: Anatomy, etiology, and technique. J. Pediatr. Surg..

[119] Pautonnier J., Goutte S., Dubourg L.D., Bacchetta J., Ranchin B., Rabilloud M., Sanlaville D. (2024). Creatinine levels in French children with Down syndrome up to ten years old. Eur. J. Pediatr..

[120] Nishino T., Endo S., Miyano H., Takemasa Y., Saito M., Umeda C., Tomii Y., Watanabe Y., Nakagawa M., Kakegawa D. (2021). Reference serum creatinine levels according to sex, age, and height in children with Down syndrome. Eur. J. Pediatr..

[121] Mariño-Ramírez L., Sharma S., Rishishwar L., Conley A.B., Nagar S.D., Jordan I.K. (2022). Effects of genetic ancestry and socioeconomic deprivation on ethnic differences in serum creatinine. Gene.

[122] Chuang G.-T., Tsai I.-J., Tsau Y.-K. (2021). Serum Creatinine Reference Limits in Pediatric Population-A Single Center Electronic Health Record-Based Database in Taiwan. Front. Pediatr..

[123] Yamakawa S., Nagai T., Uemura O. (2018). Down syndrome and mild kidney dysfunction. Pediatr. Int..

[124] Nishino T., Endo S., Miyano H., Umeda C., Tomii Y., Watanabe Y., Nakagawa M., Kakegawa D., Fujinaga S. (2021). Is the estimated glomerular filtration rate formula useful for evaluating the renal function of Down syndrome?. Pediatr. Int..

[125] Schwartz G.J., Haycock G.B., Spitzer A. (1976). Plasma creatinine and urea concentration in children: Normal values for age and sex. J. Pediatr..

[126] Schwartz G.J., Muñoz A., Schneider M.F., Mak R.H., Kaskel F., Warady B.A., Furth S.L. (2009). New equations to estimate GFR in children with CKD. J. Am. Soc. Nephrol..

[127] Uemura O., Honda M., Matsuyama T., Ishikura K., Hataya H., Yata N., Nagai T., Ikezumi Y., Fujita N., Ito S. (2011). Age, gender, and body length effects on reference serum creatinine levels determined by an enzymatic method in Japanese children: A multicenter study. Clin. Exp. Nephrol..

[128] Dilanthi H.W., Kularatnam G.a.M., Jayasena S., Jasinge E., Samaranayake D.B.D.L., Wickramasinghe V.P. (2017). Validity of the use of Schwartz formula against creatinine clearance in the assessment of renal functions in children. Sri Lanka J. Child Health.

[129] Pottel H., Delanaye P., Cavalier E. (2024). Exploring Renal Function Assessment: Creatinine, Cystatin C, and Estimated Glomerular Filtration Rate Focused on the European Kidney Function Consortium Equation. Ann. Lab. Med..

[130] Huang Y.-N., Huang J.-Y., Wang C.-H., Su P.-H. (2023). Long-Term Non-Congenital Cardiac and Renal Complications in Down Syndrome: A Study of 32,936 Patients. Children.

[131] Stone N.J., Robinson J.G., Lichtenstein A.H., Bairey Merz C.N., Blum C.B., Eckel R.H., Goldberg A.C., Gordon D., Levy D., Lloyd-Jones D.M. (2014). 2013 ACC/AHA Guideline on the Treatment of Blood Cholesterol to Reduce Atherosclerotic Cardiovascular Risk in Adults: A Report of the American College of Cardiology/American Heart Association Task Force on Practice Guidelines. Circulation.

[132] Hattori M., Fujiyama A., Taylor T.D., Watanabe H., Yada T., Park H.S., Toyoda A., Ishii K., Totoki Y., Choi D.K. (2000). The DNA sequence of human chromosome 21. Nature.

[133] Shapiro B.L. (1983). Down syndrome—A disruption of homeostasis. Am. J. Med. Genet..

[134] Pritchard M.A., Kola I. (1999). The “gene dosage effect” hypothesis versus the “amplified developmental instability” hypothesis in Down syndrome. J. Neural Transm. Suppl..

[135] Duchon A., Herault Y. (2016). DYRK1A, a Dosage-Sensitive Gene Involved in Neurodevelopmental Disorders, Is a Target for Drug Development in Down Syndrome. Front. Behav. Neurosci..

[136] Lana-Elola E., Aoidi R., Llorian M., Gibbins D., Buechsenschuetz C., Bussi C., Flynn H., Gilmore T., Watson-Scales S., Hansen M.H. (2024). Increased dosage of DYRK1A leads to congenital heart defects in a mouse model of Down syndrome. Sci. Transl. Med..

[137] Redhead Y., Gibbins D., Lana-Elola E., Watson-Scales S., Dobson L., Krause M., Liu K.J., Fisher E.M.C., Green J.B.A., Tybulewicz V.L.J. (2023). Craniofacial dysmorphology in Down syndrome is caused by increased dosage of Dyrk1a and at least three other genes. Development.

[138] Laguna A., Barallobre M.-J., Marchena M.-Á., Mateus C., Ramírez E., Martínez-Cue C., Delabar J.M., Castelo-Branco M., de la Villa P., Arbonés M.L. (2013). Triplication of DYRK1A causes retinal structural and functional alterations in Down syndrome. Hum. Mol. Genet..

[139] Blackburn A.T.M., Bekheirnia N., Uma V.C., Corkins M.E., Xu Y., Rosenfeld J.A., Bainbridge M.N., Yang Y., Liu P., Madan-Khetarpal S. (2019). DYRK1A-related intellectual disability: A syndrome associated with congenital anomalies of the kidney and urinary tract. Genet. Med..

[140] Lozic M., Minarik L., Racetin A., Filipovic N., Saraga Babic M., Vukojevic K. (2021). CRKL, AIFM3, AIF, BCL2, and UBASH3A during Human Kidney Development. Int. J. Mol. Sci..

[141] Tsygankov A.Y. (2023). TULA Proteins in Men, Mice, Hens, and Lice: Welcome to the Family. Int. J. Mol. Sci..

[142] Tsygankov A.Y. (2020). TULA proteins as signaling regulators. Cell Signal.

[143] Tsygankov A.Y. (2018). TULA-family proteins: Jacks of many trades and then some. J. Cell Physiol..

[144] Yuen R.K., Neumann S.M., Fok A.K., Peñaherrera M.S., Mcfadden D.E., Robinson W.P., Kobor M.S. (2011). Extensive epigenetic reprogramming in human somatic tissues between fetus and adult. Epigenetics Chromatin.

[145] Henriksen K.J., Chang A., Bayliss G.P. (2018). Kidney Transplant Outcomes in 2 Adults With Down Syndrome. Kidney Int. Rep..

[146] Ohki Y., Kawabe M., Yamamoto I., Kobayashi A., Kanzaki G., Koike K., Ueda H., Tanno Y., Urabe F., Miki J. (2023). Early Recurrence of Immunoglobulin A Nephropathy after Kidney Transplantation in a Patient with Down Syndrome. Nephron.

[147] Edvardsson V.O., Kaiser B.A., Polinsky M.S., Baluarte H.J. (1995). Successful living-related renal transplantation in an adolescent with Down syndrome. Pediatr. Nephrol..

